# Cultivar-Dependent Responses in Plant Growth, Leaf Physiology, Phosphorus Use Efficiency, and Tuber Quality of Potatoes Under Limited Phosphorus Availability Conditions

**DOI:** 10.3389/fpls.2021.723862

**Published:** 2021-08-30

**Authors:** Leangsrun Chea, Ana Meijide, Catharina Meinen, Elke Pawelzik, Marcel Naumann

**Affiliations:** ^1^Division of Quality of Plant Products, Department of Crop Sciences, University of Goettingen, Goettingen, Germany; ^2^Division of Agronomy, Department of Crop Sciences, University of Goettingen, Goettingen, Germany

**Keywords:** ATP, cultivars, phosphorus deficiency, phosphorus efficiency, leaf, potato, sugars, tuber quality

## Abstract

The limited availability of phosphorus (P) in soils causes a major constraint in the productivity of potatoes, which requires increased knowledge of plant adaptation responses in this condition. In this study, six potato cultivars, namely, Agria, Lady Claire, Milva, Lilly, Sieglinde, and Verdi, were assessed for their responses on plant growth, leaf physiology, P use efficiency (PUE), and tuber quality with three P levels (P_low_, P_med_, and P_high_). The results reveal a significant variation in the cultivars in response to different P availabilities. P-efficient cultivars, Agria, Milva, and Lilly, possessed substantial plant biomass, tuber yield, and high P uptake efficiency (PUpE) under low P supply conditions. The P-inefficient cultivars, Lady Claire, Sieglinde, and Verdi, could not produce tubers under P deprivation conditions, as well as the ability to efficiently uptake P under low-level conditions, but they were efficient in P uptake under high soil P conditions. Improved PUpE is important for plant tolerance with limited P availability, which results in the efficient use of the applied P. At the leaf level, increased accumulations of nitrate, sulfate, sucrose, and proline are necessary for a plant to acclimate to P deficiency-induced stress and to mobilize leaf inorganic phosphate to increase internal PUE and photosynthesis. The reduction in plant biomass and tuber yield under P-deficient conditions could be caused by reduced CO_2_ assimilation. Furthermore, P deficiency significantly reduced tuber yield, dry matter, and starch concentration in Agria, Milva, and Lilly. However, contents of tuber protein, sugars, and minerals, as well as antioxidant capacity, were enhanced under these conditions in these cultivars. These results highlight the important traits contributing to potato plant tolerance under P-deficient conditions and indicate an opportunity to improve the P efficiency and tuber quality of potatoes under deficient conditions using more efficient cultivars. Future research to evaluate molecular mechanisms related to P and sucrose translocation, and minimize tuber yield reduction under limited P availability conditions is necessary.

## Introduction

Potatoes demand high phosphorus (P) availability in soils because their roots are relatively inefficient in P uptake with low soil P concentration (Sandaña, 2016; Wacker-Fester et al., 2019). P fertilizers have been applied to increase soil P and tuber yield. However, up to 80% of the applied P turns into insoluble complex and organically bound forms, which are not readily available for plants (Shi et al., 2019). The unavailable P tends to remain in the upper soil layers and tends to flow toward surface water, which causes eutrophication (King et al., 2015). Considering environmental concerns along with a decrease in globally reserved P resources (Cordell and White, 2014), improved agronomic practices that reduce P fertilizer input are necessary to enhance P efficiency for the production of potatoes.

For potatoes, P use efficiency (PUE) can be defined as the capability of the plants to produce biomass or tuber yield per unit of applied P (Veneklaas et al., 2012). Under low P availability conditions, PUE is strongly influenced by P uptake efficiency (PUpE), the amount of total plant P uptake per unit of applied P (Sandaña, 2016). Plants respond to limited P availability through morphological and physiological adjustments to acclimate to deficient-P conditions. The previous study reported increased root biomass compared with the shoots of potato (cv. Milva) under P-deficient conditions, which indicates preferential photoassimilate allocation to roots to enhance P uptake (Chea et al., 2021). However, root sugar concentrations were reduced by 50–80% under these conditions. Based on these results, additional investigation on leaf biochemical alterations under P-deficient conditions is required to explain the shortage in photoassimilates for translocation. Owing to the indispensable role of P in energy transfer, marginal P deficiency could reduce adenosine triphosphate (ATP) synthesis for consumption in the Calvin cycle, causing a reduction in total photosynthetic rates (Carstensen et al., 2018; Dixon et al., 2020). Electron transport and ATP synthesis mediation in the Calvin cycle is important to increase photosynthesis (Simkin et al., 2019). Furthermore, a large amount of inorganic P (P_i_) in leaf tissues has to be generated through internal P recycling to maintain its concentration in the stroma for carbon fixation (Wissuwa et al., 2005). Therefore, efficient use of plant internal P for photosynthesis is essential to ensure sufficient photoassimilates for shoot growth and translocation. However, Wissuwa et al. (2005) and Fredeen et al. (1989) showed an accumulation of soluble sugars under P-deficient conditions, which suggested that the utilization of photoassimilates for plant growth was restricted. In maize, Plénet et al. (2000) reported a direct effect of P deficiency on growth rather than on leaf photosynthesis. Little is known about potatoes with respect to photosynthesis and leaf biochemical alteration induced by P deficiency. In addition, the stress caused by P deficiency triggers the accumulation of osmolytes, such as proline, for stress detoxification (Hayat et al., 2012) and greater uptake of essential minerals, such as nitrogen (N) and sulfur (S) (Chea et al., 2021). These adaptation mechanisms are necessary for plant viability under P-deficient conditions. Therefore, potato cultivars with enhanced P use efficiency and photosynthetic capacity, under limited P availability conditions, could be an alternative strategy to overcome situations of P deficiency.

P availability in soils highly influences potato tuber yield and quality (Naumann et al., 2020). However, besides the above-mentioned morphological and biochemical responses, the impact of limited P availability on potato tuber quality is less documented, except for a few reports on tuber yield, dry matter, starch, protein, and sugars (Öztürk et al., 2010; Fernandes et al., 2015; Leonel et al., 2017). Wang and Frei (2011) showed an increased concentration of micronutrients, protein, and antioxidant capacity in potato tubers and grains of various crops as a result of nutrient uptake alteration and modulation of key enzymes under abiotic stress conditions. Moreover, the recent discovery showed an increase in leaf minerals and antioxidant compounds, such as total flavonoids and total phenolics, in response to P deficiency (Chea et al., 2021); thus, we can hypothesize that P deficiency may also stimulate the uptake of minerals and the antioxidant capacity in tubers.

In this study, we sought to assess (I) the impact of P deficiency on plant growth, PUE, photosynthetic characteristics, and leaf biochemical properties, and (II) the effects of P deficiency on the quality of potato tubers. The goals of this study are to identify P-efficient cultivars and to provide further insights into the tolerant mechanisms of potatoes under P deficiency conditions. We also provided the first report on the implications of P deficiency on ascorbic acid and the antioxidant capacity of potato tubers.

## Materials and Methods

### Plant Materials

Six potato cultivars were used, which consisted of four table potatoes, Agria, Milva, Lilly, and Sieglinde, and two processing potatoes, Lady Claire and Verdi. Lady Claire was obtained from Meijer Potato (Rilland, the Netherlands); Agria, Lilly, and Sieglinde were obtained from Kartoffel Mueller (Nersingen, Germany); Milva and Verdi were obtained from Europlant Pflanzenzucht GmbH (Lüneburg, Germany). These cultivars were selected based on their popularity for production in the respective regions, and their differences in morphological and yield characteristics. Additionally, Sieglinde is known for its limited biomass and tuber yield production, and it is a cultivar sensitive to nutrient deficiency (Mauromicale et al., 2006). The description of each cultivar is shown in Supplementary Table 1.

### Experimental Setup and Crop Management

The experiment was performed under outdoor conditions using six cultivars and three P levels (0.02, 0.2, and 1.2 g kg^−1^ soil; designated as P_low_, P_med_, and P_high_, respectively) with four replications. The average temperature during this period (June–September 2019) was 19 ± 6.5°C. Diurnal photosynthetic active radiation, temperature, relative humidity, and daily precipitation are shown in Supplementary Figure 1. The sandy soil used in the experiment had a pH of 4.8 and extractable calcium acetate lactate P (CAL-P) of 0.06 g kg^−1^ soil. To lower P concentration, the soil was mixed with equal amounts of medium-sized quartz sand, so that all the treatments had the same initial soil P. Afterward, the different P treatments were induced by applying Ca(H_2_PO_4_)_2_ basally as powder. The application of other nutrients is shown in Supplementary Table 2. Soil Ca concentration was balanced by the addition of CaCO_3_. After all the nutrients were applied, soil pH was in the range of 5.5–7. Then, the soil mixture, with a bulk density of 1.1 kg dm^−3^, was filled in 6 L pots (Mitscherlich, STOMA, Emmingen-Liptingen, Germany). Afterward, a single germ bud of 1–2 cm was taken from each potato seed using a ball shaper and was planted in each pot at a depth of 5 cm. These procedures were adapted from Koch et al. (2019) and Wacker-Fester et al. (2019). All the seedlings were germinated within 10 days after they were planted. Water was supplied regularly to maintain optimum soil moisture based on visual observation.

### Plant Growth Assessment, Leaf Gas Exchange Measurements, and Sample Preparation

Plant height development was monitored during the experiment. Leaf gas exchange measurements were conducted on the terminal leaf of a fully developed leaflet at the fourth position from the top using a portable photosynthesis system (LI-6800, LI-COR Biosciences, Lincoln, NE, United States) after 35, 53, and 70 days after emergence (DAE). Prior to the measurements, each plant was adapted for 1 h under constant environmental conditions (temperature = 20°C, relative humidity = 60%, and photosynthetic photon flux density [PPFD] = 400 μmol m^−2^ s^−1^ at plant level) in a climate chamber. For the measurement, the CO_2_ concentration, relative humidity, and leaf temperature inside the cuvette (4 cm^2^) were adjusted to 400 ppm, 50%, and 25°C, respectively. Light response curves were generated by starting with a PPFD of 1,400 μmol m^−2^ s^−1^ in 13 steps (180 s/step) to zero. Based on light response curve measurements, the CO_2_ assimilation rate, stomatal conductance, and intercellular CO_2_ concentration measured at PPFD of 400 μmol m^−2^ s^−1^ were compared for different cultivars and P treatments at different stages of development.

The whole leaflet at the fourth position from the top was taken 53 DAE after leaf gas exchange measurements for mineral and biochemical analyses. A part of the sample was freshly ground with liquid nitrogen and stored at −20°C. The plants were mature, and the tubers were fully developed at 87 DAE; therefore, the whole plants were harvested and partitioned into shoots, roots, and tubers. Another part of the fresh tuber was used for ascorbic acid determination, immediately after cutting, as described below. The sub-samples of each plant part were freeze-dried in a freeze dryer (EPSILON 2-40, Christ, Osterode am Harz, Germany) for 4 days. The biomass of the plant was then calculated as a combination of total shoots (including sampled young leaves 53 DAE) and root dry matter (DM). Dry samples were ground in a hammer mill (DFH 48 Culatti, Kinematica, Malters, Switzerland) with a 0.5 mm sieve.

### Plant Mineral, Ion, and Residual Soil P Analyses

Minerals in the leaves, shoots, roots, and tubers were analyzed by extracting 100 mg of the freeze-dried sample according to Koch et al. (2019) to determine the concentration of P, S, potassium (K), calcium (Ca), magnesium (Mg), manganese (Mn), iron (Fe), and zinc (Zn). These mineral concentrations were determined by inductively coupled plasma optical emission spectrometry (ICP-OES) (Varian, Palo Alto, CA, United States). Shoot, root, and tuber P contents were calculated by multiplying the P concentration of the respective plant part with its DM content. Total P uptake of each plant was calculated as the combination of shoot, root, and tuber P contents. Afterward, P uptake efficiency (PUpE) and P use efficiency (PUE) was calculated based on Sandaña (2016) as follows:

(1) $$\begin{array}{l} \text{PUpE }\left(\text{mg P uptake }\text{m}{\text{g}}^{-1}\text{ applied P}\right)=\frac{\text{Total P uptake }\left(\text{mg plan}{\text{t}}^{-1}\right)}{\text{Applied P }\left(\text{mg po}{\text{t}}^{-1}\right)} \end{array}$$

(2) $$\begin{array}{l} \text{PUE }\left(\text{g tuber DM}\text{ m}{\text{g}}^{-1}\text{ applied P}\right)=\frac{\text{Tuber DM }\left(\text{g plan}{\text{t}}^{-1}\right)}{\text{Applied P }\left(\text{mg po}{\text{t}}^{-1}\right)} \end{array}$$

The applied P of each treatment was determined by multiplication of the applied P concentration with the soil dry weight per pot. Carbon (C) and N concentration in the leaves and tubers were analyzed from 0.75 g of the freeze-dried samples using the dry combustion method with a Vario EL analyzer (Elementar, Langenselbold, Germany). Phosphate (${\text{PO}}_{4}^{3-}$), nitrate (${\text{NO}}_{3}^{-}$), and sulfate (${\text{SO}}_{4}^{2-}$) in the leaves and tubers were determined by extracting 20 mg of the freeze-dried samples with 1 ml of 0.1 M HCl, and the extracts were analyzed with an ion chromatography system (ECO IC, Metrohm, Herisau, Switzerland) in accordance with the procedures described by Koch et al. (2020). The concentration of each ion was expressed as mg of the respective mineral per unit of sample DM. After the harvest, available P in the soil was extracted following the Olsen method using a bicarbonate solution (Hartmann et al., 2019), and was determined in accordance with molybdenum blue procedures (Murphy and Riley, 1962) with a UV-Vis spectrophotometer (HP 8453, Hewlett Packard, Böblingen, Germany) at 882 nm absorbance.

### Leaf Chlorophyll, Free Proline, ATP, and Protein Analyses

Leaf chlorophyll and free proline were extracted by homogenizing 20 mg of freshly ground samples with 250 μl of 80% ethanol at 95°C. The mixture was then centrifuged at 10,600 *g* for 10 min to collect the supernatant. The procedures were sequentially repeated twice with 150 μl of 80% ethanol and 150 μl of 50% ethanol. The supernatants from each step were pooled and measured for leaf chlorophyll and free proline concentration according to Koch et al. (2019) and Chea et al. (2021), respectively. For ATP and protein measurements, 100 mg of the fresh leaf samples were extracted with 1 ml of cold 5% trichloroacetic acid for 5 min, and the mixture was centrifuged for 10 min at 13,000 *g* at 4°C to collect the supernatant. The ATP concentration in the supernatant was determined using an ATPlite assay kit (PerkinElmer, Waltham, MA, United States) in accordance with the instructions of the manufacturer. The pellet from the sample extract was resuspended with 400 μl of 0.1 M NaOH for 30 min at 95°C. After centrifugation for 10 min at 10,000 *g*, protein concentration was determined using a Bradford protein kit (Merck, Darmstadt, Germany) based on the modified methods of Zor and Selinger (1996). Bovine serum albumin was used as the standard.

### Sugar Analyses of Leaves and Tubers

Soluble sugars in the leaves were extracted by homogenizing 50 mg of the freeze-dried samples with 700 μl of 80% acetonitrile in a shaker at 420 rpm for 3 h. Then, 50 μl of 3.6% K_4_[Fe(CN)_6_]^*^3H_2_O and 50 μl of 7.2% ${\text{ZnSO}}_{4}^{*}$7H_2_O were subsequently added to precipitate proteins, followed by 30 min of centrifugation at 15,000 *g* to collect the supernatant, and it was stored at −20°C. For the tubers, 0.75 g of each freeze-dried sample was extracted with 3 ml of distilled water by shaking for 1 h at 420 rpm. Protein precipitation was executed by sequentially adding 0.5 mL of 3.6% K_4_[Fe(CN)_6_]^*^3H_2_O and 0.5 ml of 7.2% ${\text{ZnSO}}_{4}^{*}$7H_2_O in the mixture, which was then subjected to 20 min of centrifugation at 2,600 *g* to collect the supernatant. The extraction procedures were repeated two times without protein precipitation. The supernatants from each step were pooled and filled up to 10 ml with distilled water and were stored at −20°C. For measurement, the extract was thawed and centrifuged for 30 min at 15,000 *g*. The supernatant was then filtered through a 0.45-μm membrane with the help of a 13 mm syringe (VWR, Darmstadt, Germany). Finally, 20 μl of the filtered extract was used for the quantification of soluble sugars (sucrose, glucose, and fructose) by high-performance liquid chromatography (Jasco, Pfungstadt, Germany). As eluent, 80% acetonitrile was used through a 5 μm column (LiChrospher 100 NH2, Merck, Darmstadt, Germany) at 22°C and with a flow rate of 1 ml min^−1^.

### Tuber Quality Analyses

The starch concentration of the tubers was determined by a polarimetric method according to the procedures of Koch et al. (2019). Tuber N concentration was converted to crude protein concentration with a factor of 6.25 (AOAC, 2005). The ascorbic acid content of the potato tubers was analyzed based on a 2,6-dichlorophenolindophenol (DIP) titrimetric method described in Sonntag et al. (2020).

To determine total phenolics (TPC), total flavonoids (TFC), and antioxidant capacity, 100 mg of the freeze-dried tuber sample was extracted two times with 1 ml of 99.9% methanol. The supernatants were combined and filled up to 2 ml with methanol. TPC and TFC of the extract were analyzed according to Chea et al. (2021). Antioxidant capacity was determined based on 2,2-diphenyl-1-picrylhydrazyl (DPPH) assay, and Trolox equivalent antioxidant capacity (TEAC) assay based on Kaur et al. (2013), with slight modifications. For the DPPH assay, 20 μl of the extract was suspended with 180 μl of 0.2 mM DPPH. After incubation for 30 min in the dark, the mixture was read with a plate reader (Synergy HTX, Biotek, Winooski, VT, United States) at 515 nm absorbance. The TEAC assay is based on the ability of antioxidants to scavenge 2,2′-azino-bis-3-ethylbenzothiazoline-6-sulphonic acid (ABTS) radical cations. For measurement, 10 μl of the extract was mixed with 150 μl of the ABTS working solution, containing 0.15 mM of ABTS and 0.5 mM of K_2_S_2_O_8_. The mixture was incubated at room temperature for 10 min in darkness, and the absorbance was read with a plate reader at 734 nm. In both the assays, 99.9% of methanol was used as a negative control, and radical scavenging capacity was determined based on the difference in the negative control and sample extract absorbance. The antioxidant capacity was calculated against the Trolox standard calibration curve and expressed as μmol Trolox equivalent (TE) g^−1^ DM.

### Statistical Analysis

Data of plant growth, mineral concentration, P efficiency, leaf biochemical properties, and tuber quality parameters were subjected to two-way ANOVA. Tukey's honestly significant difference (HSD) test at *p* < 0.05 was performed for pairwise comparisons among the P treatments when there were significant differences in ANOVA. The association among the observed traits was assessed by Pearson's correlation. These analyses were conducted following the methods of Gomez and Gomez (1984) using the Statistix 8.0 software (Analytical Software, Tallahassee, United States). Graphical presentations were prepared in Sigmaplot 12.5 (Systat Software, San Jose, CA, United States).

## Results

### Plant Growth and Tuber Yield

**Figure 1:**
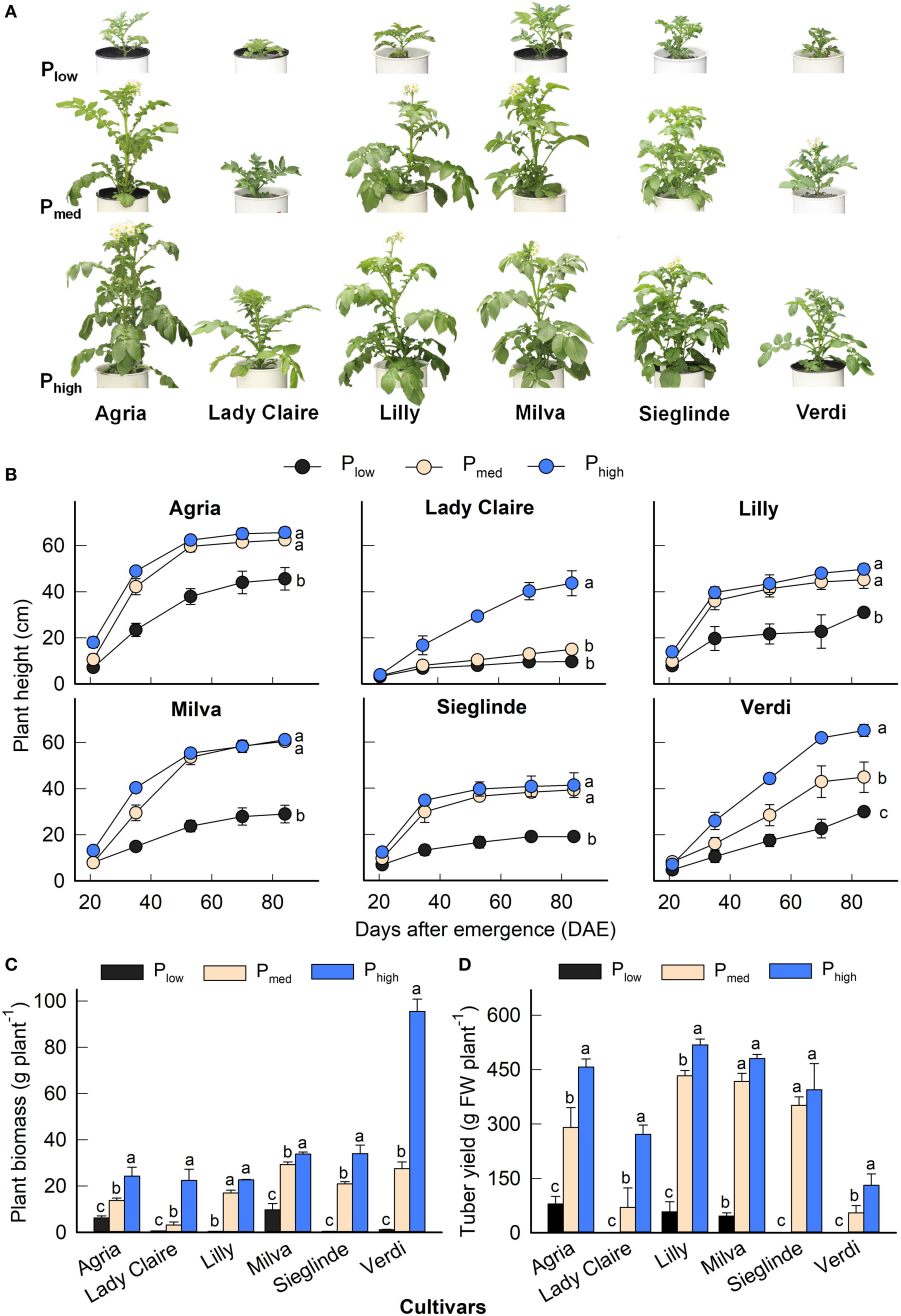
**(A)** Shoot phenotype 40 days after emergence (DAE), **(B)** plant height development, **(C)** plant biomass, and **(D)** tuber yield of potato cultivars with the application of P_low_, P_med_, and P_high_. Letters in lowercase indicate a significant difference between P treatments of each cultivar by Tukey's honestly significant difference (HSD) test at *p* < 0.05. Error bar represents SE of means (*n* = 4). FW, fresh weight.

The P applications had a significant effect on plant height, plant biomass, and tuber yield for all the cultivars (Figure 1). The effects of the cultivars and the interaction among the cultivars and P levels were also significant (Supplementary Table 3). The plant height of each cultivar evolved at different rates during the growing period depending on the P application (Figures 1A,B). The plant biomass of Lady Claire, Lilly, Sieglinde, and Verdi was strongly inhibited with the P_low_ treatment (0.40–1.13 g plant^−1^), and it increased by 24- to 85-fold under higher P availability conditions. Agria and Milva had relatively greater plant biomass (6.16 and 9.70 g plant^−1^, respectively) than the other cultivars under P_low_ conditions, which also increased in response to higher P levels (Figure 1C). However, under P_low_ conditions, Lady Claire, Sieglinde, and Verdi were not able to produce tubers, while the tuber yield of Agria, Lilly, and Milva ranged from 45.90 to 79.42 g plant^−1^ (Figure 1D). The variations in mean values of plant height, plant biomass, and tuber yield among the cultivars and P treatments are shown in Supplementary Table 4.

### Tissue P Concentration, PUE, and Residual Soil P

**Figure 2:**
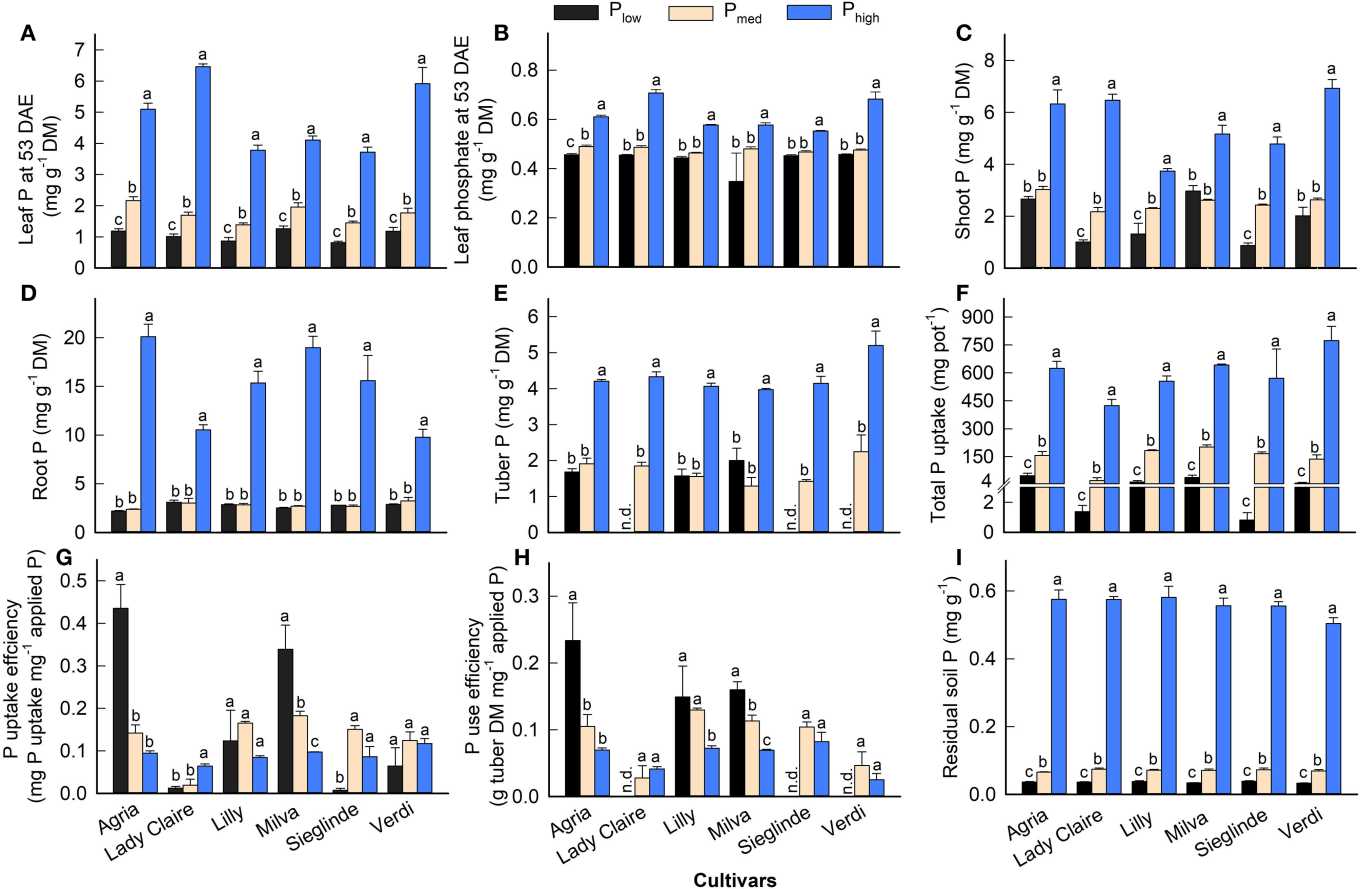
**(A–E)** Phosphorus (P) concentration in different plant parts, **(F)** total P uptake, **(G)** P uptake efficiency, **(H)** P use efficiency, and **(I)** residual soil P concentration of potato cultivars with the application of P_low_, P_med_, and P_high_. P uptake efficiency, amount of total P uptake per unit of applied P, and P use efficiency, tuber dry matter production per unit of P uptake. Phosphate concentration **(B)** is expressed as mg of mineral (P) per unit of DM. Different letters in lowercase indicate a significant difference between P treatments of each cultivar by Tukey's HSD test at *p* < 0.05. Error bar represents SE of means (*n* = 4). DM, dry matter; n.d., not determined because of no tuber production.

Mineral analyses of leaves 53 DAE revealed low P concentrations under P_low_ conditions, which ranged from 0.82 (Sieglinde) to 1.19 mg g^−1^ (Agria) (Figure 2A; Supplementary Table 4). The application of P_med_ and P_high_ increased leaf P by 50–83% and 3- to 5-fold, respectively, compared with P_low_. We also observed a reduction of leaf phosphate under P_low_ (Figure 2B), but it was at a lower magnitude than that of leaf P. The P_low_ treatment reduced shoot P concentrations, but there was no significant difference between P_low_ and P_med_ in root and tuber P concentration (Figures 2C–E). Low P availability in the soil suppressed total P uptake, but it enhanced the PUpE of Agria and Milva by 44% (Figures 2F,G). In contrast, the PUpE of Lady Claire, Sieglinde, and Verdi was hampered by low soil P. Furthermore, PUE remained highest for Agria among the cultivars under P_low_ conditions (Figure 2H). We were unable to determine the PUE of Lady Claire, Sieglinde, and Verdi under P_low_ conditions due to the absence of tubers; thus, we assumed a very low PUE for these cultivars under low P availability conditions. The analysis of soil P after harvest, for which the Olsen extraction method was used, revealed that soil P concentration under P_high_ conditions was 14–16 times and 7–9 times higher than that under P_low_ and P_med_ conditions, respectively (Figure 2I).

**Figure 3:**
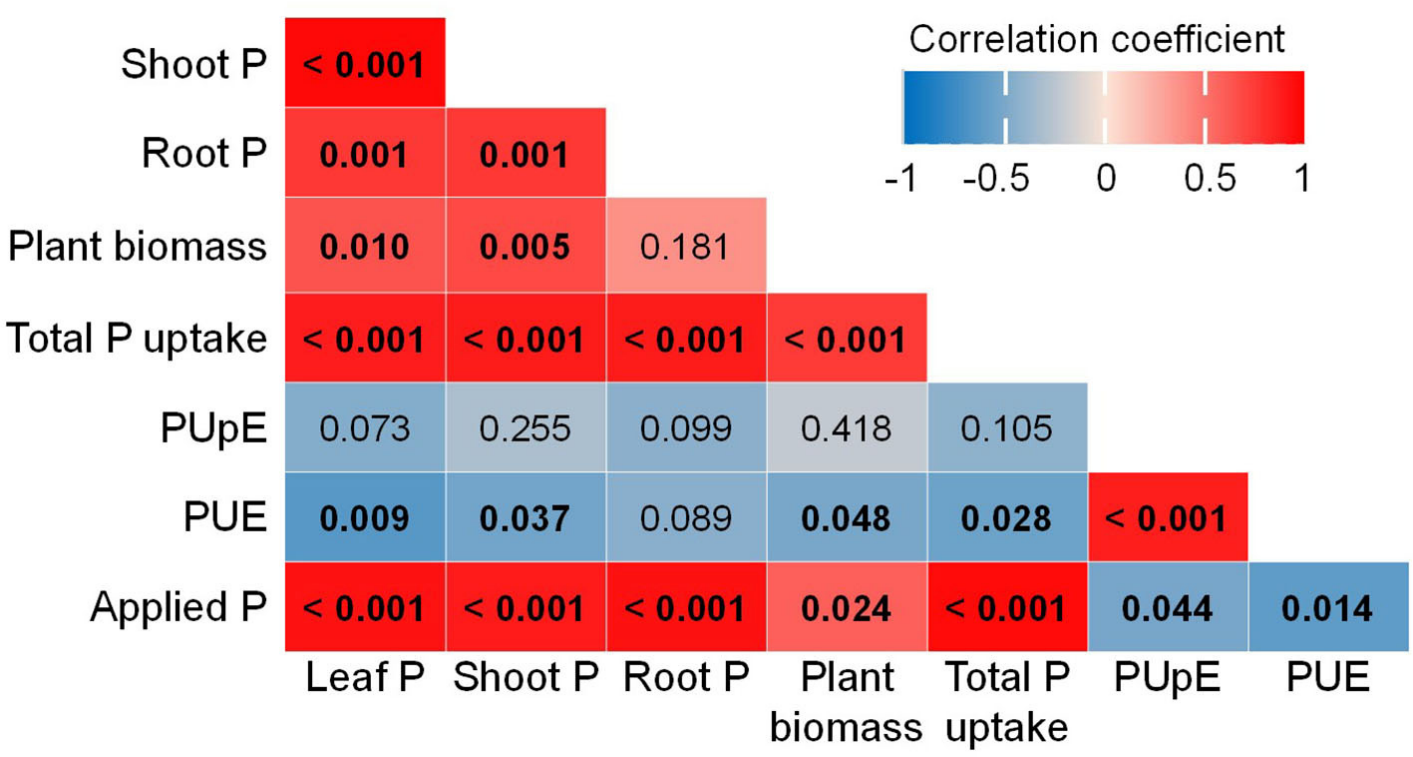
Correlation among plant P concentration, biomass, total P uptake, P uptake efficiency (PUpE), P use efficiency (PUE), and P applications (applied P). Color gradients represent Pearson's correlation coefficient, and values in bold of each cell indicate *p*-values which are significant at < 0.05.

Furthermore, the Pearson's correlations of traits associated with plant P concentration and PUE show positive correlations among leaf P, shoot P, plant biomass, total P uptake, and applied P levels. However, these parameters negatively correlated with PUE, which was positively associated with PUpE (Figure 3).

### Leaf Minerals and Ions

In response to the P_low_ treatment, leaf essential minerals and ions such as N, nitrate, S, and sulfate significantly increased compared with higher P availability (Table 1). Furthermore, there were significant correlations between leaf N and S with their respective ionic forms (Supplementary Figure 2). The impact of P deficiency on other leaf macro and micronutrients is shown in Supplementary Table 5.

**Table 1:** Leaf concentration (mg g^−1^ dry matter, DM) of nitrogen (N), nitrate, sulfur (S), and sulfate of potato cultivars with P_low_, P_med_, and P_high_.

	**Cultivars**					
	**Agria**	**Lady Claire**	**Lilly**	**Milva**	**Sieglinde**	**Verdi**
**N**						
P_low_	57.23 ±1.00 a	56.87 ±0.91 a	54.66 ±1.24 a	56.25 ±0.82 b	58.12 ±3.87 a	57.47 ±4.85 a
P_med_	52.40 ±1.61 ab	45.83 ±0.60b	45.90 ±2.02 a	53.83 ±1.50b	51.55 ±5.04a	51.26 ±3.25a
P_high_	47.27 ±1.83b	62.77 ±2.51a	42.89 ±3.74a	45.62 ±2.56a	42.33 ±0.85a	55.57 ±1.98a
**Nitrate**						
P_low_	10.46 ± 0.52a	13.52 ± 1.16a	16.33 ± 0.75a	7.31 ± 2.45a	12.50 ± 1.20a	14.31 ±1.65a
P_med_	6.06 ± 0.51b	14.67 ± 0.53a	8.16 ± 0.30b	5.92 ± 0.28b	6.74 ± 0.90b	9.14 ± 1.71b
P_high_	4.32 ± 0.19 b	9.97 ± 0.47b	6.81 ±0.20c	4.65 ±0.25c	3.44 ±0.12c	6.42 ± 0.39c
**S**						
P_low_	7.53 ± 0.73a	6.96 ± 0.23a	12.13 ± 0.75a	11.93 ± 0.99a	8.25 ± 0.24a	5.21 ± 0.39a
P_med_	4.92 ± 0.20b	7.50 ± 1.76a	5.25 ± 0.26b	5.59 ± 0.74b	4.62 ± 0.24b	3.72 ± 0.40ab
P_high_	3.98 ± 0.27c	3.85 ± 0.10b	3.63 ± 0.46c	3.87 ± 0.19c	3.05 ± 0.13c	3.56 ± 0.14b
**Sulfate**						
P_low_	5.88 ± 0.44a	5.86 ± 0.20a	8.89 ± 0.12a	6.92 ± 2.36a	6.54 ± 0.35a	4.77 ± 0.31a
P_med_	4.19 ± 0.06b	6.23 ± 1.15a	4.70 ± 0.19b	4.42 ± 0.16b	4.00 ± 0.08b	3.72 ± 0.08b
P_high_	3.80 ± 0.12b	3.70 ± 0.06b	3.76 ± 0.08c	3.87 ± 0.11b	3.54 ± 0.05b	3.60 ± 0.05b

*Nitrate and sulfate concentrations are expressed as mg of respective minerals per unit of DM. Mean values ± SE (n = 4) with different letters in lowercase indicating significant difference between P treatments of each cultivar by Tukey's honestly significant difference (HSD) test at p < 0.05*.

### Leaf Gas Exchange and Leaf Biochemical Characteristics

**Figure 4:**
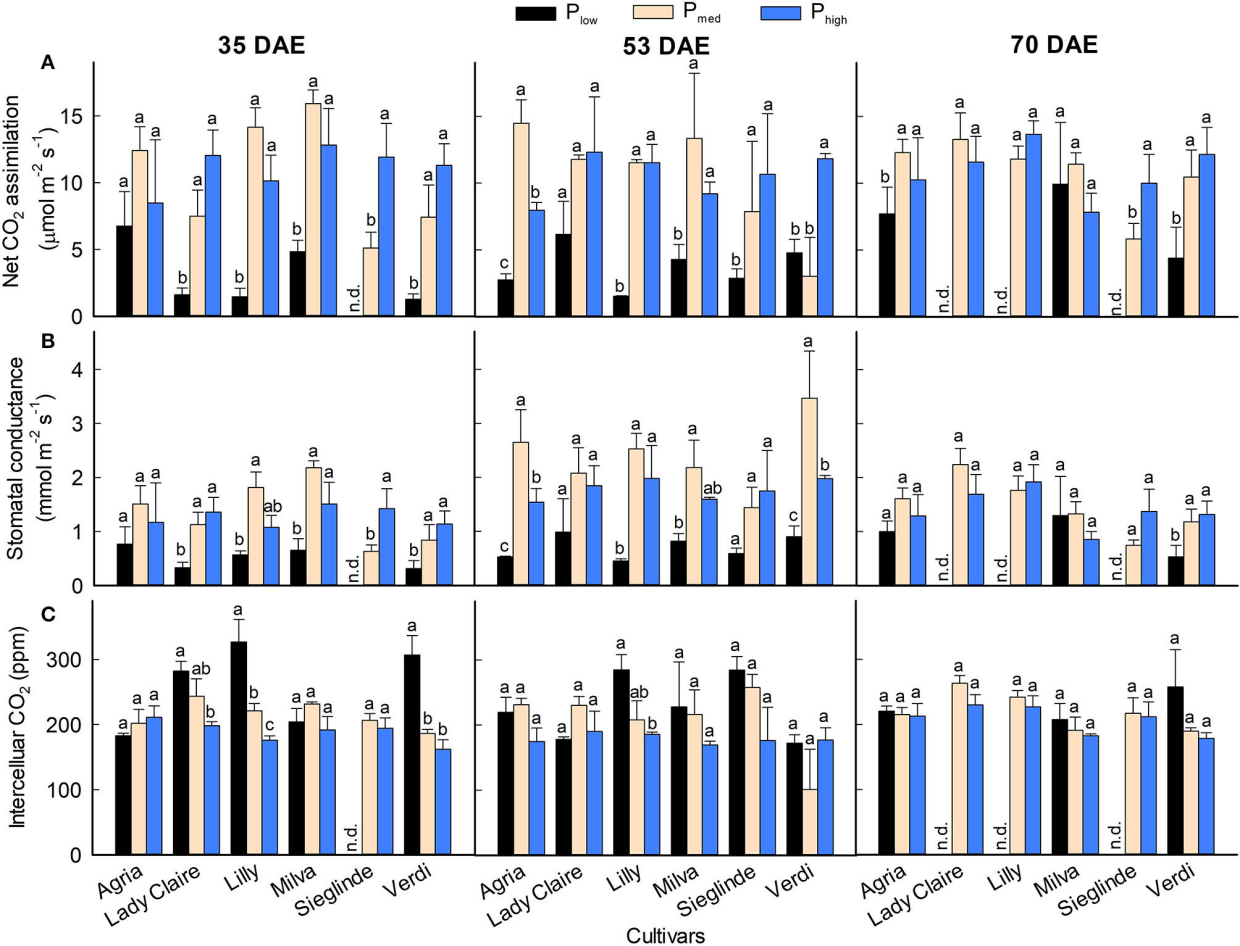
**(A)** CO_2_ assimilation, **(B)** stomatal conductance, and **(C)** intercellular CO_2_ concentration of potato cultivars with the application of P_low_, P_med_, and P_high_. Different letters in lowercase indicate a significant difference between P treatments of each cultivar by Tukey's HSD test at *p* < 0.05. Error bar represents SE of means (*n* = 2–3); n.d., not determined because of too small leaf area to fit in the cuvette.

CO_2_ assimilation rate and stomatal conductance were significantly affected by the cultivars and P levels on all measurement dates (Figures 4A,B; Supplementary Table 3). In general, both parameters were reduced by 13–90% on all measurement dates under P_low_ conditions compared with P_med_. Across the measurement dates, the CO_2_ assimilation and stomatal conductance of Agria and Milva were relatively higher than those of the other cultivars under P_low_ conditions. We observed an increasing trend in the CO_2_ assimilation and stomatal conductance of Lady Claire, Sieglinde, and Verdi under P_high_ conditions compared with P_med_, but they were either stable or reduced by 16–45% for Agria, Lilly, and Milva. The influence of P applications on intercellular CO_2_ concentration was to a variable extent in regard to cultivars and measurement dates (Figure 4C). For Lady Claire, Lilly, Sieglinde, and Verdi, the intercellular CO_2_ concentration was enhanced under P_low_ conditions on at least one of the measurement dates.

**Figure 5:**
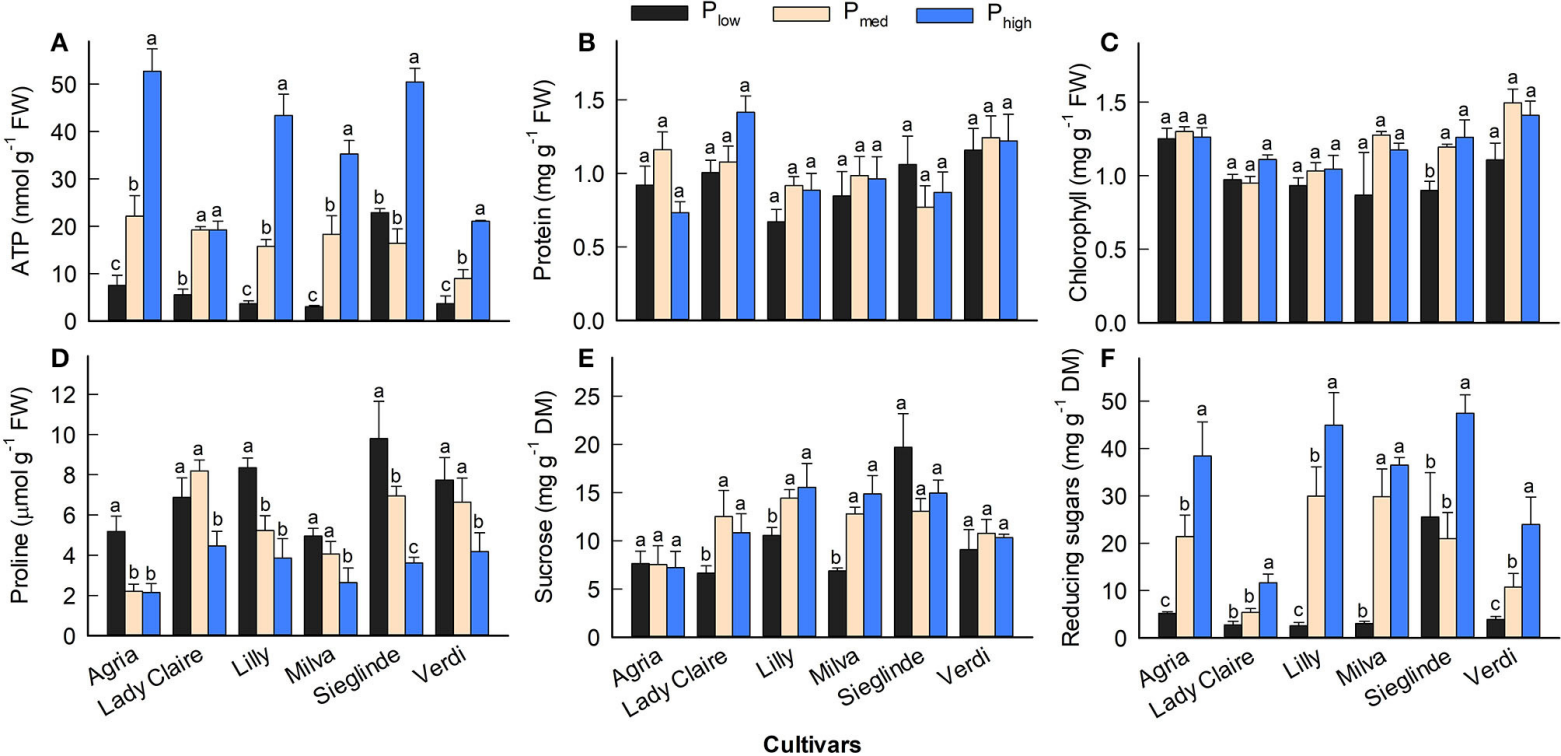
**(A–F)** Leaf biochemical characteristics of potato cultivars 53 DAE with P_low_, P_med_, and P_high_. Different letters in lowercase indicate a significant difference between P treatments of each cultivar by Tukey's HSD test at *p* < 0.05. Error bar represents SE of means (*n* = 4). FW, fresh weight; DM, dry matter.

**Figure 6:**
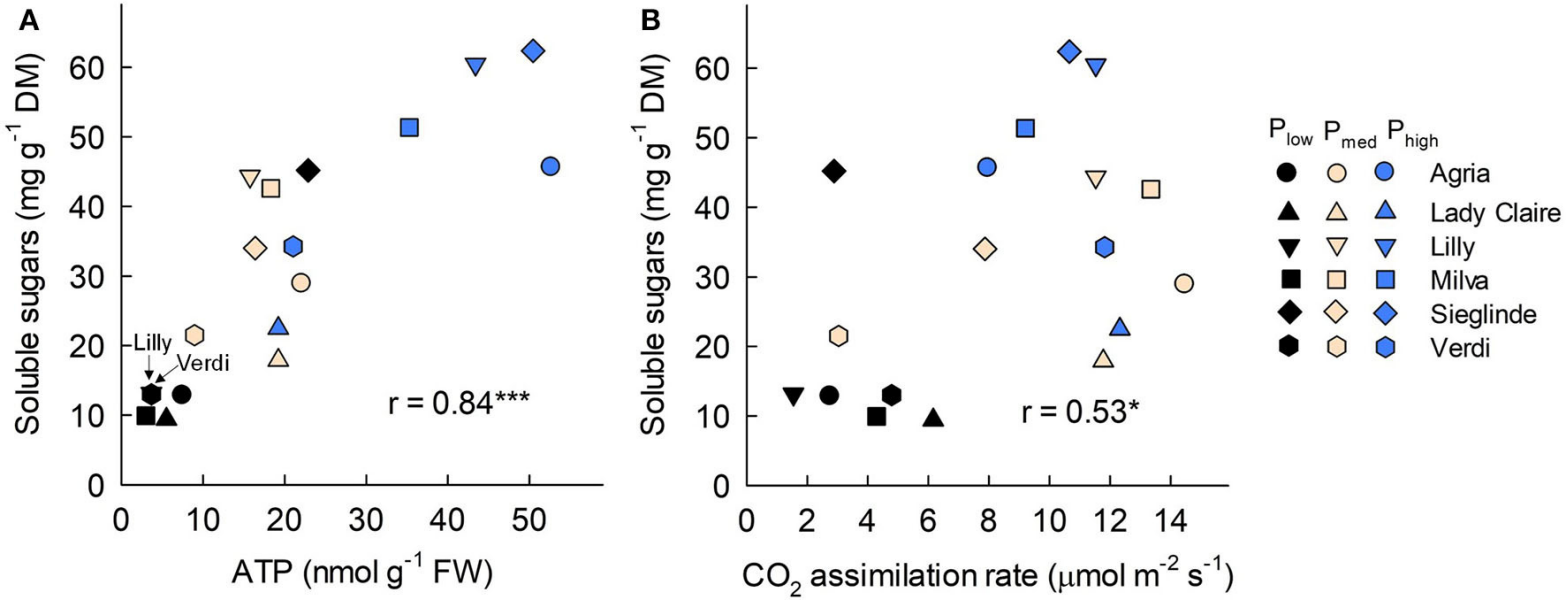
Correlation among leaf soluble sugars with **(A)** adenosine triphospate (ATP) concentration and **(B)** CO_2_ assimilation rate measured 53 DAE. FW, fresh weight; DM, dry matter; * and *** indicate significant correlation at *p* < 0.05 and *p* < 0.001, respectively.

Furthermore, the analyses of leaves sampled 53 DAE show that leaf ATP ranged from 3.02 (Milva) to 22.87 nmol g^−1^ (Sieglinde) under P_low_ conditions and that it was enhanced by 2- to 12-fold under P_high_ conditions (Figure 5A; Supplementary Table 6). Leaf protein and chlorophyll concentrations of each cultivar were less affected by P applications (Figures 5B,C). However, leaf proline under P_low_ conditions ranged from 5.10 (Agria) to 9.80 μmol g^−1^ (Sieglinde). At a higher P application (P_high_), leaf proline was reduced by 14–63% (Figure 5D; Supplementary Table 6). Even though P availability in the soil had less influence on leaf sucrose in all the cultivars, except for Lady Claire and Milva, reducing sugars (fructose and glucose) were significantly increased in response to higher P supply (Figures 5E,F). In addition, total soluble sugars positively correlated with ATP concentration and CO_2_ assimilation, although the correlation coefficient between the total soluble sugars and CO_2_ assimilation was moderate (Figure 6).

### Tuber Quality

**Figure 7:**
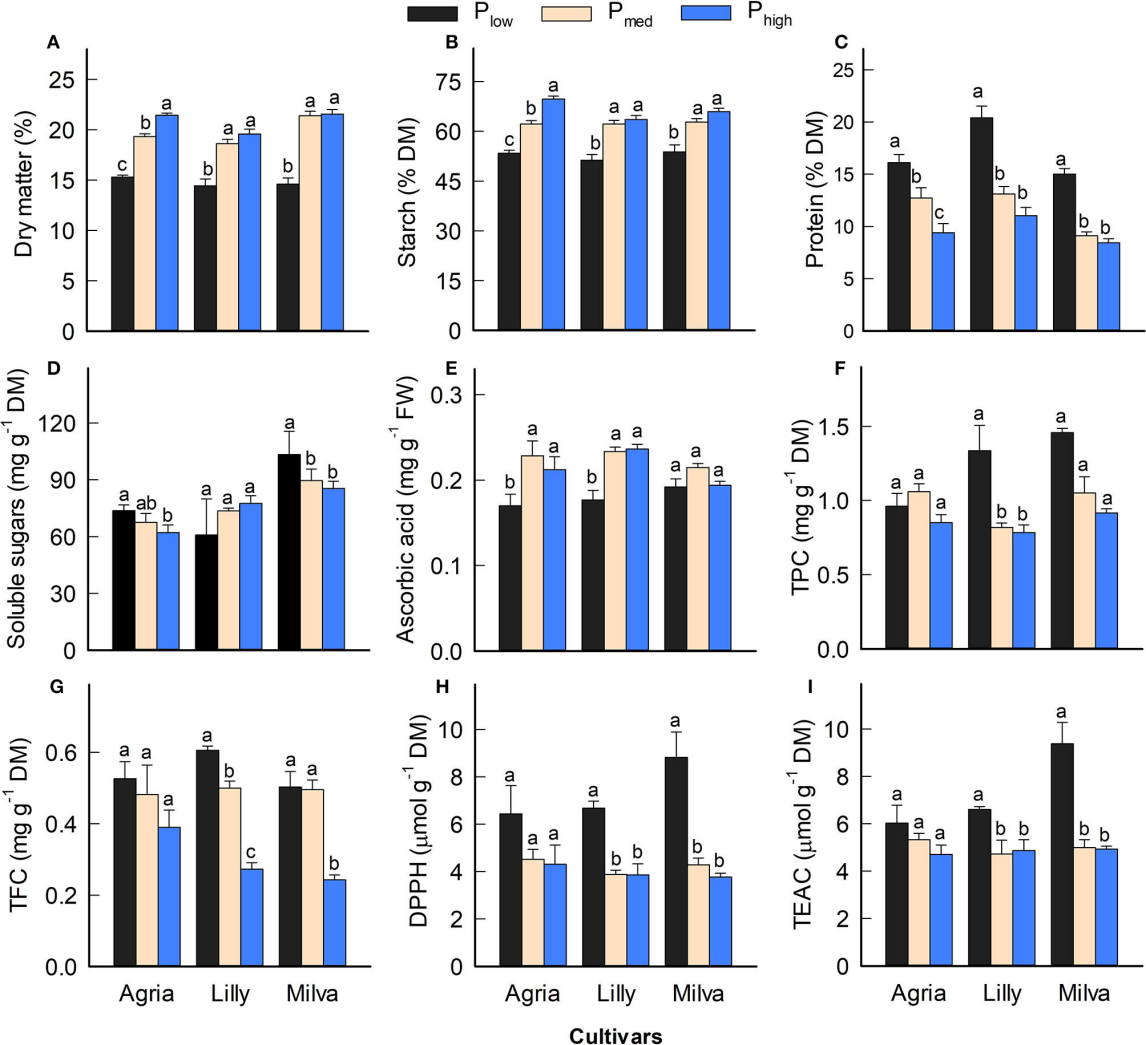
**(A–I)** Tuber quality characteristics of potato cultivars with the application of P_low_, P_med_, and P_high_. Different letters in lowercase indicate a significant difference between P treatments of each cultivar by Tukey's HSD test at *p* < 0.05. DM, dry matter; FW, fresh weight; TPC, total phenolic concentration; TFC, total flavonoid concentration; DPPH, antioxidant capacity by 2,2-diphenyl-1-picrylhydrazyl assay; TEAC, Trolox equivalent antioxidant capacity.

Since Lady Claire, Sieglinde, and Verdi were unable to produce tubers under P_low_ conditions, the results are demonstrated only for Agria, Lilly, and Milva to assess the impact of P_low_ on tuber quality characteristics. The significant effects of the cultivars, P levels, and their interactions were observed in many quality parameters (Supplementary Table 7). Figure 7 shows that P_low_ reduced tuber DM by 22–32%, starch concentration by 14–23%, and ascorbic acid by 10–25% compared with both P_med_ and P_high_. However, tuber protein was increased under P_low_ conditions by 27–64% and 71–85% compared with P_med_ and P_high_, respectively. Tuber soluble sugars of Agria and Milva under P_low_ conditions were higher than those under higher P levels; however, the soluble sugars of Lilly were not significantly different between the P treatments. There was no significant difference in TPC and TFC among the P applications of Agria, but increasing the P application resulted in decreasing TPC and TFC for Lilly and Milva. Furthermore, the antioxidant capacity (DPPH and TEAC) of P_low_ was 11–57% higher than that of P_med_ and P_high_. Besides these tuber quality characteristics, the P applications also affected mineral and ion concentrations to variable extents (Table 2). Although P_low_ reduced tuber C and phosphate concentration by 2–40%, it increased the concentrations of the other minerals and ions (K, Ca, Mg, S, Cu, Fe, Mn, Zn, nitrate and sulfate by 20–85% compared with P_high_.

**Table 2:** Tuber mineral and ion concentration of potato cultivars with P_low_, P_med_, and P_high_.

	**Cultivars**		
	**Agria**	**Lilly**	**Milva**
**Phosphate** (mg g^−1^ DM)			
P_low_	0.98 ± 0.06b	0.71 ± 0.09b	1.22 ± 0.14b
P_med_	0.91 ± 0.08b	0.66 ± 0.06b	0.61 ± 0.10b
P_high_	1.86 ± 0.07a	1.86 ± 0.07a	2.36 ± 0.10a
**Nitrate** (mg g^−1^ DM)			
P_low_	6.49 ± 0.47a	10.77 ± 0.95a	10.66 ± 1.17a
P_med_	0.66 ± 0.61b	3.03 ± 0.60b	1.34 ± 0.83b
P_high_	0.74 ± 0.53b	1.58 ± 0.67b	0.88 ± 0.83b
**C** (mg g^−1^ DM)			
P_low_	408.31 ± 2.02b	399.20 ± 5.15b	403.51 ± 1.53
P_med_	417.99 ± 2.61a	419.77 ± 3.25a	418.34 ± 1.08
P_high_	419.52 ± 2.26a	412.17 ± 3.64ab	417.23 ± 1.08
**K** (mg g^−1^ DM)			
P_low_	31.73 ± 0.39 a	32.27 ± 1.06a	32.87 ± 0.95a
P_med_	16.91 ± 0.51b	18.43 ± 0.67b	16.63 ± 0.67b
P_high_	15.22 ± 0.44c	16.03 ± 0.75b	15.07 ± 0.67b
**Ca** (mg g^−1^ DM)			
P_low_	2.18 ± 0.14a	2.83 ± 0.29a	4.56 ± 0.58a
P_med_	0.55 ± 0.18b	1.00 ± 0.18b	0.74 ± 0.41b
P_high_	0.72 ± 0.15b	0.85 ± 0.20b	0.79 ± 0.41b
**Mg** (mg g^−1^ DM)			
P_low_	1.71 ± 0.06a	1.53 ± 0.08a	1.76 ± 0.14a
P_med_	1.25 ± 0.08b	1.29 ± 0.05b	0.94 ± 0.10b
P_high_	1.06 ± 0.07b	1.18 ± 0.06b	0.89 ± 0.10b
**S** (mg g^−1^ DM)			
P_low_	3.12 ± 0.04a	3.66 ± 0.19 a	4.23 ± 0.51a
P_med_	2.37 ± 0.06b	2.72 ± 0.12 b	2.22 ± 0.36b
P_high_	1.74 ± 0.05c	2.11 ± 0.13c	1.87 ± 0.36b
**Sulfate** (mg g^−1^ DM)			
P_low_	2.66 ± 0.10a	3.63 ± 0.68a	3.44 ± 0.58a
P_med_	2.81 ± 0.22a	2.70 ± 0.19a	1.94 ± 0.10b
P_high_	1.63 ± 0.06b	1.69 ± 0.05b	1.53 ± 0.08b
**Fe** (μg g^−1^ DM)			
P_low_	48.70 ± 5.11a	46.70 ± 9.46a	77.50 ± 9.80a
P_med_	35.60 ± 6.60ab	31.00 ± 5.98 a	37.50 ± 7.60b
P_high_	22.50 ± 5.72b	52.50 ± 6.69a	62.50 ± 7.60ab
**Mn** (μg g^−1^ DM)			
P_low_	20.40 ± 1.02a	27.70 ± 2.11a	22.40 ± 3.51a
P_med_	9.30 ± 1.31b	12.30 ± 1.33 b	7.00 ± 2.48b
P_high_	9.50 ± 1.14b	12.50 ± 1.48 b	10.80 ± 2.48ab
**Zn** (μg g^−1^ DM)			
P_low_	47.10 ± 1.47a	65.00 ± 3.65a	55.00 ± 6.45a
P_med_	38.10 ± 1.90b	45.00 ± 2.31b	30.00 ± 4.56b
P_high_	32.50 ± 1.65b	35.00 ± 2.58c	40.00 ± 4.56ab

*Phosphate, nitrate, and sulfate concentrations are expressed as mg of respective minerals per unit of dry matter, DM. Mean values ± SE (n = 4) with different letters in lowercase indicate a significant difference between P treatments of each cultivar by Tukey's HSD test at p < 0.05*.

## Discussion

A pot experiment was conducted to elucidate the responses of the six potato cultivars to soil P levels (P_low_, P_med_, and P_high_). Under pot conditions, the availability of applied P for the plants is less interfered with by P immobilization and buffering capacity of the soils, which usually occur under field conditions. The results provide a further understanding of the impacts of P deficiency and the tolerance mechanisms of potato cultivars, from plant to leaf levels.

### Differential Responses in Growth and PUE of Potato Cultivars to P Availability

The highest plant height, plant biomass, and tuber yield were seen under P_high_ conditions, which indicates that in the experiments there was sufficient P in soils with P application at 1.2 g kg^−1^. All the cultivars exhibited deficiency under P_low_ conditions through stagnated height development, reduced plant biomass, and less or no tuber yield formation (Figure 1). The reduction in plant biomass under P-deficient conditions was at a magnitude greater than that reported in pot studies by Lee et al. (2013) and Wacker-Fester et al. (2019), which suggested that P-deficient conditions in this pot study were severe. The variation in plant biomass and tuber yield of potato cultivars was also documented (Lee et al., 2013; Soratto et al., 2015; Wacker-Fester et al., 2019), which allowed us to identify P-efficient cultivars. In potatoes, tuber yield is important for classifying the P responsiveness of cultivars (Soratto et al., 2015); thus, it is an excellent parameter that indicates the tolerance of cultivars to P deficiency. In this study, the P-efficient cultivars, Agria, Milva, and Lilly, produced substantial tuber yield, but the P-inefficient cultivars, Lady Claire, Sieglinde, and Verdi, were unable to produce tubers under P-deficient conditions. Furthermore, Lady Claire, Sieglinde, and Verdi maintained substantial tissue P concentration and plant biomass, and they had relatively high PUpE and PUE under P-deficient conditions (Figures 1, 2A–**H**). The ranges of PUpE and PUE were similar to those in Sandaña (2016) under field conditions. In contrast, although Lady Claire, Sieglinde, and Verdi had similar plant P concentrations compared with the other cultivars, they were characterized by very low plant biomass, low PUpE, and low PUE under P-deficient conditions. This indicates that these cultivars conserved the sparingly available P in the plant for viability, and they might lack traits associated with enhanced P uptake and allocation for growth and tuber formation. The separation of cultivars for their efficiency under limited P availability conditions is also confirmed based on the principal component analysis of traits associated with P efficiency, as shown in Supplementary Figure 3. Although residual P availability in soils under P-deficient conditions was similar among the cultivars, the enhanced P uptake of P-efficient cultivars may have been caused by improved root traits, which could modify the amount of P available for plants (Lee et al., 2013). Therefore, improved PUpE is important to enhance PUE under limited P supply conditions (Wang et al., 2010). The increased PUpE and PUE of Lady Claire, Sieglinde, and Verdi under high P availability conditions suggests that these cultivars are efficient in P uptake when soil P availability is not a limiting factor. Increased P availability in soil resulted in high plant P concentration, P uptake, and plant biomass (Figure 3), but it cannot give assurance for enhanced PUE if the PUpE is low. Consequently, high residual soil P, after harvest, implies inefficient use of P fertilizer, which, under field conditions, is a potential risk for the environment (Heuer et al., 2017).

### Importance of Leaf Minerals and Ions in Mitigating P Deficiency

In plants, P exists as inorganic orthophosphate forms (${\text{PO}}_{4}^{3-}$ or P_i_) and organic phosphate esters (Veneklaas et al., 2012). The results reveal a lesser reduction in leaf P_i_ compared with leaf P under P-deficient conditions, which could be caused by an increase in a P_i_ fraction under these conditions. Under P-deficient conditions, plants sense cytosolic P_i_ shortage through recycling the entire P in the vacuole, to increase P_i_ concentration for efflux into cytosol and chloroplast (Shen et al., 2011; Long et al., 2019). Therefore, P translocation to active photosynthetic tissue and internal P recycling could be adaptations of potato cultivars under stress conditions induced by P deprivation. Furthermore, leaf nitrate also increased under P-deficient conditions. The mechanisms underlying nitrate accumulation have not been thoroughly understood; however, they could be caused by nitric oxide generation due to oxidative stress acclimation under P-deficient conditions (Fu et al., 2018). High nitric oxide production was also reported on soybean leaves (Ramos-Artuso et al., 2019) and *Arabidopsis* (Royo et al., 2015) under P deprivation conditions. In the complex nitrate cycle in plants, nitrate is required for nitric oxide biosynthesis, and the turnover of nitric oxide also produces nitrate. This complete cycle is regulated by nitrate reductase (Astier et al., 2018). Furthermore, nitrate assimilation is an energetically costly process (Nunes-Nesi et al., 2010), which could be inhibited under P-deficient conditions, resulting in an accumulation of nitrate in leaves. Additionally, the uptake and allocation of other N forms such as ammonium and amides may be altered under P-deficient conditions, as implied by the relationship between leaf N and nitrate shown in Supplementary Figure 2. Furthermore, leaf sulfate concentration may also increase to fulfill the S demand of sulfolipid generation for replacing phospholipid under P-deficient conditions and to balance leaf anion-to-cation ratio under low phosphate ion conditions (Misson et al., 2005; Rouached, 2011).

### Leaf Photosynthesis and Biochemical Adaption in Response to P Deficiency

Besides the alterations in leaf minerals and ions, P deficiency reduced stomatal conductance and CO_2_ assimilation rate at different magnitudes (Figures 4A,B). Reduction in photosynthesis under P-deficient conditions was also observed in barley (Carstensen et al., 2018) and soybean (Singh and Reddy, 2015). P deficiency suppresses photosynthesis rate through disruption on electron transport and reduction in ATP and NADPH synthesis (Carstensen et al., 2018). In this study, although fast photosynthesis measurements were conducted at 180 s at each radiation level, it was sufficient to obtain steady-state results, because the PPFD inside the cuvette for the data used in this study and under ambient conditions, before the measurements, was the same. The results further reveal that at least two of the three P-efficient cultivars (viz. Agria and Milva) had relatively high CO_2_ assimilation rates and stomatal conductance, especially 35 and 70 DAE, which explained the substantial shoot biomass production and tuber yield of these cultivars under P starvation conditions. The minimal disruption in the photosynthesis of these cultivars under P-deficient conditions could be caused by improved P allocation to leaves to increase the leaf area. In soybean, Chaudhary et al. (2008) also showed the importance of P allocation to shoots to increase PUE and leaf area under P deprivation conditions. However, the reduction of CO_2_ assimilation in P-efficient cultivars under high P supply conditions might be linked to their high leaf area (Figure 1A). Leaves of the plants expand in response to high P supply, causing high leaf area (Shi et al., 2019), and eventually, become thinner. Therefore, photosynthetic machinery per unit of leaf area may be reduced. However, increased leaf area under high P supply conditions could compensate for the reduction in photosynthesis because of a greater light interception.

Although leaf photosynthesis was reduced by P deficiency, leaf protein and chlorophyll were less affected (Figures 5B,C). In leaves, the majority of N is present in chlorophyll and proteins of the thylakoids (Perchlik and Tegeder, 2018); therefore, in this study, the accumulation of leaf N may contribute to the maintenance of protein and chlorophyll in leaves under P-deficient conditions. A similar observation in maize indicated that leaves of P-deficient plants are less associated with a reduction in chlorophyll concentration because these leaves eventually become thicker and appear to be bluish-green (Plénet et al., 2000). However, the results reveal that P deficiency also modulates leaf ATP, sucrose, reducing sugars, and proline at different levels depending on the cultivar. Since the P applications did not significantly affect leaf protein concentration (Figures 5B,C), the effects of P levels on ATP and proline could be compared and discussed based on per leaf fresh weight. At the leaf level, P plays an essential role as a substrate for ATP synthesis in the chloroplast (Carstensen et al., 2018). Even though a chloroplast P transporter (*AtPHT4;1*) is proposed to mediate chloroplast Pi for ATP synthesis activities under limited P supply conditions in *Arabidopsis* (Karlsson et al., 2015), in this study, sufficient chloroplast P_i_ may have not been maintained under severe P-deficient conditions, which ultimately results in ATP reduction. ATP limitation under P-deficient conditions hampers the use of NADPH in the Calvin cycle (Carstensen et al., 2018); thus, CO_2_ assimilation is reduced for sugar production.

In this study, P deficiency also influenced sugar metabolism. There was no significant reduction in leaf sucrose concentration in many of the cultivars under P-deficient conditions (Figure 5E). This could be due to a high cleavage of sucrose to fructose and glucose under high P availability conditions, but under P-deficient conditions, the conversion of sucrose was inhibited. This resulted in a huge increase of these reducing sugars in response to increasing P applications, especially for Agria, Lilly, and Milva (Figure 5F). The conversion of sucrose to reducing sugars is regulated by enzymes such as invertase, sucrose phosphate synthase, and fructose 1,6-bisphosphatase, which are enhanced under sufficient P supply conditions (García-Caparrós et al., 2021). The less or no reduction of sucrose in the leaves under P-deficient conditions could also be caused by either limited sugar transport or the sugar conservation response of plants. Under P-deficient conditions, plants maintain large amounts of compatible non-toxic solutes such as sucrose and proline for cellular osmotic adjustment, stabilizing cell structure, and scavenging free radicals (Ashraf and Foolad, 2007; Hayat et al., 2012). In *Arabidopsis*, a high sucrose level is important to induce P starvation-responsive genes for mobilizing plant internal P (Lei et al., 2011). Under stress conditions induced by P deficiency, proline is synthesized from glutamate in the cytosol; however, depending on the recovery from stress, proline is rapidly oxidized into glutamate, while ATP is also generated during this oxidation process to maintain leaf viability (Launay et al., 2019). Among the cultivars, we found that Sieglinde had a relatively high ATP, proline, sucrose, and total reducing sugar (fructose and glucose) concentration compared with the other cultivars under P-deficient conditions. These results indicate a high stress intensity of this cultivar under P-deficient conditions, which may have inhibited sugar translocation. It also recommends further investigations on sucrose transporters that may explain molecular mechanisms underlying the relatively high sucrose of these cultivars under low P availability conditions. In this study, there was a positive correlation between the concentration of leaf total soluble sugars and the concentration of ATP (Figure 6A). The correlation between total soluble sugars and CO_2_ assimilation was also positively significant (Figure 6B), but it was to a lesser extent, which suggested that improved CO_2_ assimilation is important for sugar production, provided that there is a sufficient amount of ATP in the reaction to convert the assimilated CO_2_ into sugars.

### Impacts of P Deficiency on Tuber Quality

We present the implications of P deficiency on the tuber quality of potato in addition to plant agronomic and biochemical characteristics as outlined above. P is involved in several key enzymes that regulate the starch synthesis, and it is also a key element of starch composition (Nielsen et al., 1994; Naumann et al., 2020). Although we did not observe a significant difference in tuber P concentration between P_low_ and P_med_, a reduction of P application resulted in a significant decrease in tuber DM and starch concentration (Figures 7A,B). This suggests a compromise between improving PUE and the quality parameters. The tuber DM of all the cultivars under P-deficient conditions was also below the range (16–18%) of many potatoes available in the market (Storey, 2007), which may have a significant impact on the market acceptability. However, protein concentration was enhanced under low P availability conditions (Figure 7C), which could be caused by increased N uptake as indicated by high leaf N concentration (Table 1). These results are similar to those reported by Leonel et al. (2017). However, Fernandes et al. (2015) and Öztürk et al. (2010) did not observe significant differences in tuber DM, starch, and protein concentrations between low and high soil P availability. The inconsistency of these results might be due to cultivar differences in DM and starch concentration. Furthermore, the P-deficient condition reported in those studies might be less severe, compared with this study, and therefore did not alter the DM, starch, and protein concentrations in the tubers. The sugar concentration of the tubers is also important for the fresh market (Storey, 2007). In this study, P deficiency enhanced sugar concentration in the tubers for Agria and Milva, but it had no significant effect on those of Lilly (Figure 7D). This indicates that under P efficiency conditions, the limited carbohydrates allocated into the tubers might be converted into sugars rather than starch. Similar to the findings of this study, Xing et al. (2020) reported a non-significant relationship between soil P availability and total soluble sugars in tubers, because some potato tubers grown under low soil P conditions also contain a relatively high sugar concentration.

The analyses of ascorbic acid, antioxidant capacity, and minerals of the potato tubers revealed a reduction of ascorbic acid in all the cultivars except for Milva, and an increase in TPC, TFC, DPPH, TEAC, minerals, and ions under P-deficient conditions (Figures 7E–I; Table 2). The concentration of ascorbic acid under different P treatments was within the ranges (0.10–0.25 mg g^−1^ FW) of potatoes in the markets (Storey, 2007), which implies that the reduction of ascorbic acid under P deficiency could be neglected. Ascorbic acid accounts for about 13% of the total antioxidant capacity of tubers (Storey, 2007). Therefore, about 20% reduction in ascorbic acid under P-deficient conditions could be compensated by an increase in other antioxidants, such as TPC and TFC, while increasing the total antioxidant capacity significantly under these conditions. Although tuber sample extraction for antioxidant capacity measurement was conducted using methanol, which aimed for hydrophilic antioxidants (Kaur et al., 2013), these antioxidants contribute the most to the total antioxidant capacity of potato tubers (Andre et al., 2007). The accumulation of these antioxidants is caused by oxidative stress induced by P deficiency, which triggers numerous plant response reactions for antioxidant systems (Wang and Frei, 2011). The increased concentration of minerals in the leaves also contributed to improving the concentration of these minerals and ions in tubers. However, nitrate concentration in tubers under P-deficient conditions exceeded the general range (<200 mg kg^−1^ FW) for potato, based on the classification of vegetables, according to nitrate concentration published by Santamaria (2006). High nitrate concentration could be caused by a higher proportion of tuber skin when the size of tubers is small under P-deficient conditions (data not shown). Increased concentrations of protein, minerals, and ions, except P and phosphate, under P-deficient conditions, could also be a result of tuber yield reduction; thus, the concentration is less diluted by the shortage of carbohydrates (Wang and Frei, 2011). The increased concentrations of tuber phytochemicals and minerals are valuable to promote the health and physical well-being of consumers (Andre et al., 2007; Wang and Frei, 2011).

## Conclusion

There is a significant variation in plant responses to P deficiency that exists among the tested potato cultivars. We could identify the P-efficient cultivars, Agria, Milva, and Lilly, possessing substantial plant biomass, tuber yield, and high PUpE under low P supply conditions, and they may be suitable for production under limited P conditions. The P-inefficient cultivars, Lady Claire, Sieglinde, and Verdi, lacked the efficiency for P uptake and the ability to produce tubers under P-deficient conditions. However, these cultivars may be efficient in P uptake at high P availability in the soils, which leads to a reduction of P loss in the environment. In response to the low supply of P, potato plants attempted to maintain essential ions (such as phosphate, nitrate, and sulfate) and compatible solutes (such as proline and sucrose) to improve internal PUE and acclimate stress, induced by P deficiency. Leaf photosynthesis also decreased under P-deficient conditions, which was associated with ATP reduction, and further resulted in reduced plant biomass and tuber yield. Furthermore, even though P deficiency significantly reduced tuber DM, concentrations of sugars and minerals, and antioxidant capacity were enhanced for the P-efficient cultivars, which can contribute to better nutritional properties of potatoes. These results indicate a possibility to improve P efficiency and tuber quality of potatoes under P-deficient conditions using P-efficient cultivars. Therefore, in the future, focus on evaluating molecular mechanisms related to P and sucrose transporters will increase the knowledge of internal PUE under limited P availability conditions.

## Data Availability Statement

The original contributions presented in the study are included in the article/Supplementary Material, further inquiries can be directed to the corresponding author.

## Author Contributions

LC performed the experiment, analyzed the data, and drafted the manuscript. CM and AM contributed to photosynthetic measurements and data analyses and revised the manuscript. EP and MN supervised, designed the experiment, and revised the manuscript. All the authors read and approved the final version of the manuscript.

## Conflict of Interest

The authors declare that the research was conducted in the absence of any commercial or financial relationships that could be construed as a potential conflict of interest.

## Publisher's Note

All claims expressed in this article are solely those of the authors and do not necessarily represent those of their affiliated organizations, or those of the publisher, the editors and the reviewers. Any product that may be evaluated in this article, or claim that may be made by its manufacturer, is not guaranteed or endorsed by the publisher.
