# Supplementary material for: Cultivar-Dependent Responses in Plant Growth, Leaf Physiology, Phosphorus Use Efficiency, and Tuber Quality of Potatoes Under Limited Phosphorus Availability Conditions
Source: Front Plant Sci. 2021 Aug 30;12:723862. doi: 10.3389/fpls.2021.723862 (PMC8435887; doi:10.3389/fpls.2021.723862)
Supplement: Supplementary file 1 [file Data_Sheet_1.pdf]

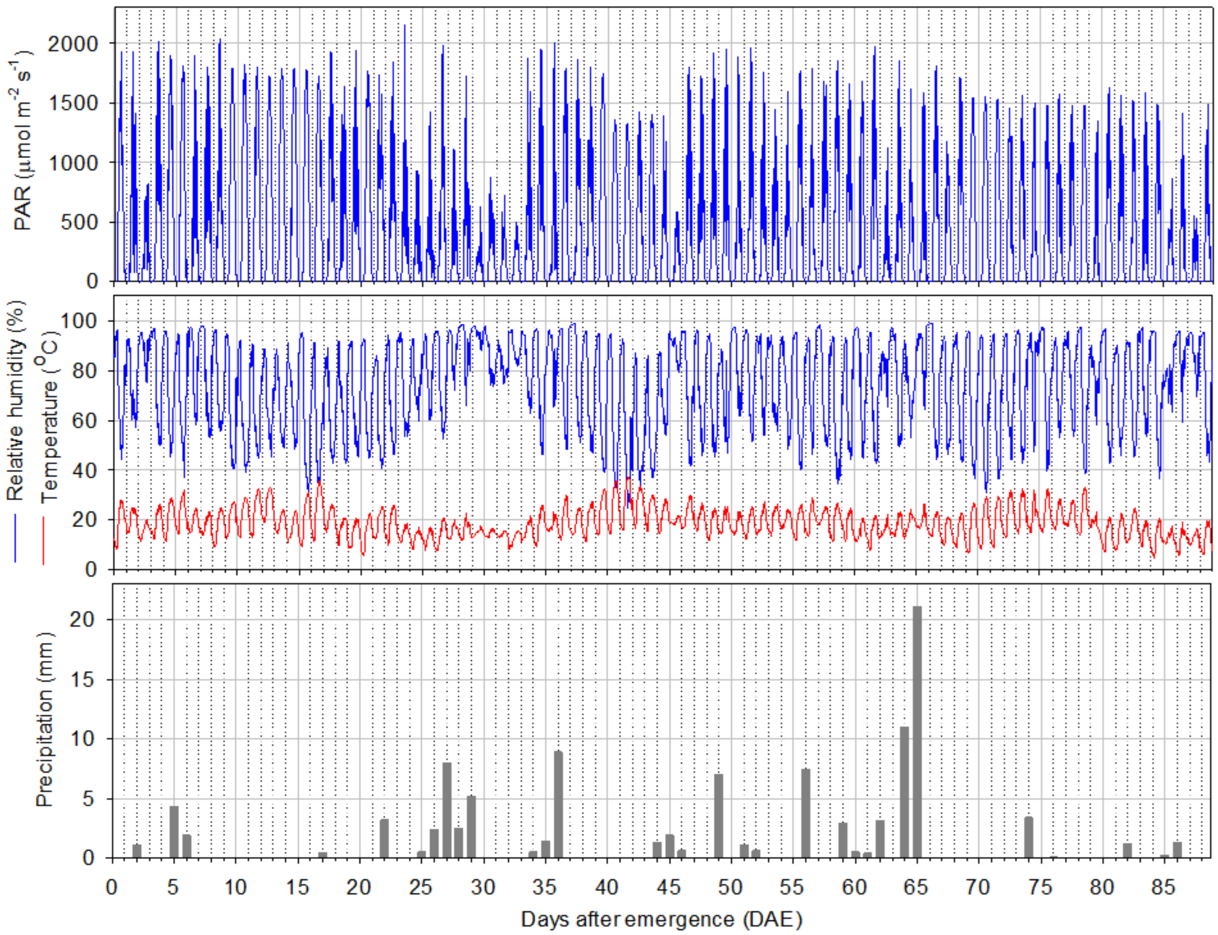

Supplementary Figure 1. Diurnal photosynthetic active radiation (PAR), temperature, relative humidity, and daily precipitation during the experiment (17 June to 12 September 2019). (Data source: Experimental Botanical Garden, University of Goettingen)

Supplementary Table 1. Description of potato cultivars

Cultivars	Maturity	Tuber skin color	Tuber dry matter	Destination
Agria	Medium	Yellow	Medium	Ware potato, convenience products, french fries, flakes, crisps
Lady Claire	Medium-late	White	High	French fries, crisps
Lilly	Early-medium	Yellow	Medium	Ware potato convenience products
Milva	Medium	Yellow	Medium	Ware potato, convenience products
Sieglinde	Early	Yellow	Medium	Ware potato, convenience products
Verdi	Medium	Yellow	High	Crisps

The further information of each cultivar can be found in the variety catalogues below:
Lilly: https://www.solana.de/kartoffelsorten-detail_en/items/lilly.html?pdf=create&id=24
Agria and Milva:
https://www.europlant.biz/fileadmin/user_upload/Brosch%C3%BCren/Europlant_A6_Varieties_E_2018-19.pdf
Lady Claire: https://www.meijerpotato.co.uk/our-varieties/crisping/lady-claire/
Sieglinde: http://heritagepotato.ca/heritage-potatoes/profiles/sieglinde/;
https://www.europotato.org/varieties/view/Sieglinde-E
Verdi: https://www.solana.de/kartoffelsorten-detail_en/items/verdi.html

Supplementary Table 2. Concentration of nutrients applied in the soil

Nutrients	Concentration (mg kg^{-1} soil)	Nutrient sources
N[1]	300; 500	$Ca(NO_3)_2 * 4H_2O$
K	330	K_2SO_4
Ca	1300	$CaCO_3$, $Ca(NO_3)_2*4H_2O$
S	250	K_2SO_4, $MgSO_4*7H_2O$, $CuSO_4*5H_2O$, $ZnSO_4*7H_2O$, $MnSO_4*H_2O$
Mg	100	$MgSO_4*7H_2O$
Cu	0.002	$CuSO_4*5H_2O$
EDTA-Fe	0.003	$C_{10}H_{12}FeN_2NaO_8*3H_2O$
Zn	0.002	$ZnSO_4*7H_2O$
B	0.0006	H_3BO_3
Mo	0.002	$Na_2MoO_4*2H_2O$
Mn	0.006	$MnSO_4*H_2O$

[1]N was applied at 300 mg kg^{-1} for Agria, Lilly, Milva, and Sieglinde and at 500 mg kg^{-1} N for Lady Claire and Verdi according to the fertilizer recommendations by seed suppliers. N was added in splits, 75% basally and 25% top-dressing at 50 DAE. These nutrients were dissolved in water and applied to the soils. After all nutrients were added, soil pH of P_{low}, P_{med}, and P_{high} was in range of 6.8–7.0, 6.3–6.6, and 5.5–5.8, respectively.

Supplementary Table 3. Analysis of variance (ANOVA) of plant growth, P use efficiency, leaf minerals, and leaf biochemical characteristics of six potato cultivars under P_{low}, P_{med}, and P_{high}

**Parameters**	**ANOVA**		
	**Cultivars (C)**	**P levels (P)**	**C x P**
Plant height	***	***	***
Plant biomass	***	***	***
Tuber yield	***	***	***
P concentration			
Leaf at 53DAE	***	***	***
Shoot	***	***	***
Root	***	***	***
Tuber	***	***	***
Total P uptake	***	***	*
P uptake efficiency	***	*	***
P use efficiency	***	*	***
Leaf photosynthesis			
CO_2 assimilation 35 DAE	***	***	***
CO_2 assimilation 53 DAE	***	***	***
CO_2 assimilation 70 DAE	*	*	**
Stomatal conductance 35 DAE	*	***	**
Stomatal conductance 53 DAE	**	***	***
Stomatal conductance 70 DAE	ns	ns	***
Intercellular CO_2 35 DAE	**	*	**
Intercellular CO_2 53 DAE	*	ns	**
Intercellular CO_2 70 DAE	ns	ns	*
Leaf mineral concentration			
N	***	***	***
C	**	***	ns
K	**	***	***
S	***	***	***
Ca	***	***	**
Mg	***	***	***
Mn	***	***	***
Fe	ns	ns	ns
Zn	ns	****	ns
Leaf ions			
Phosphate	***	***	***
Nitrate	***	***	***
Sulfate	***	***	***
Leaf biochemical properties			
ATP	***	***	***
Protein	**	ns	ns
Chlorophyll	***	ns	ns
Proline	***	***	ns
Sucrose	***	***	*
Reducing sugars	***	***	***

ns, *, **, and *** indicate non-significance and significance at p<0.05, p<0.01, and p<0.001, respectively.

Supplementary Table 4. Means±SE of plant morphology, P concentration, and P use efficiency

	Cultivars					
	**Agria**	**Lady Claire**	**Lilly**	**Milva**	**Sieglinde**	**Verdi**
**Plant height (cm)**						
P_{low}	45.60±4.87	9.50±0.50	31.00±0.00	29.00±3.85	19.00±1.00	30.00±1.20
P_{med}	62.50±1.44	15.00±2.00	45.20±3.76	60.50±1.71	39.25±1.93	45.00±6.61
P_{high}	65.63±2.30	43.67±5.46	49.75±2.21	61.25±0.85	38.33±5.36	65.25±2.63
**Plant biomass (g plant^{-1})**						
P_{low}	6.16±0.95	0.59±0.04	0.41±0.03	9.70±2.73	0.40±0.06	1.14±0.17
P_{med}	13.71±1.09	3.12±1.37	16.99±1.19	29.26±1.18	20.93±0.93	27.54±2.87
P_{high}	164.88±17.95	169.46±30.75	77.60±12.63	184.80±14.17	164.78±16.49	690.47±32.16
**Tuber yield (g plant^{-1})**						
P_{low}	79.42±20.60	-	57.90±27.60	45.90±8.80	-	-
P_{med}	290.37±54.72	70.35±53.15	433.42±14.34	417.25±22.59	351.68±23.15	54.90±19.86
P_{high}	457.18±22.36	271.45±25.64	518.50±15.86	481.00±10.80	394.52±72.08	130.93±31.28
**Leaf P (mg g^{-1} DM)**						
P_{low}	1.19±0.07	1.01±0.08	0.87±0.12	1.26±0.09	0.82±0.05	1.18±0.12
P_{med}	2.17±0.12	1.70±0.10	1.39±0.06	1.96±0.13	1.45±0.06	1.77±0.16
P_{high}	5.10±0.19	6.46±0.09	3.78±0.16	4.10±0.14	3.72±0.16	5.92±0.52
**Leaf Phosphate (mg g^{-1} DM)**						
P_{low}	0.46±0.01	0.46±0.00	0.44±0.01	0.35±0.12	0.45±0.00	0.46±0.00
P_{med}	0.49±0.01	0.49±0.01	0.46±0.00	0.48±0.01	0.47±0.00	0.48±0.01
P_{high}	0.61±0.01	0.71±0.01	0.58±0.00	0.58±0.01	0.55±0.00	0.68±0.03
**Shoot P (mg g^{-1} DM)**						
P_{low}	2.66±0.10	1.01±0.08	1.32±0.41	2.97±0.21	0.87±0.10	2.02±0.33
P_{med}	3.03±0.12	2.17±0.17	2.30±0.03	2.62±0.03	2.43±0.04	2.63±0.07
P_{high}	6.32±0.54	6.47±0.23	3.74±0.10	5.16±0.34	4.78±0.27	6.93±0.34
**Root P (mg g^{-1} DM)**						
P_{low}	2.19±0.06	3.09±0.22	2.85±0.07	2.50±0.07	2.79±0.00	2.86±0.06
P_{med}	2.37±0.04	3.00±0.50	2.83±0.14	2.68±0.07	2.67±0.14	3.22±0.37
P_{high}	20.10±1.28	10.53±0.53	15.36±1.20	18.98±1.16	15.58±2.60	9.79±0.82
**Tuber P (mg g^{-1} DM)**						
P_{low}	1.68±0.09	-	1.57±0.19	2.00±0.34	-	-
P_{med}	1.91±0.16	1.85±0.10	1.55±0.09	1.28±0.24	1.42±0.05	2.24±0.47
P_{high}	4.21±0.05	4.33±0.14	4.07±0.09	3.97±0.03	4.15±0.19	5.20±0.40
**Total P uptake (mg pot^{-1})**						
P_{low}	15.46±2.01	0.97±0.09	0.69±0.04	28.52±7.85	0.32±0.06	2.37±0.45
P_{med}	40.71±2.75	7.46±3.47	39.16±2.46	76.45±2.47	50.87±2.26	75.54±7.97
P_{high}	24.22±3.87	22.41±4.86	22.67±0.19	33.79±0.88	34.00±3.67	95.46±5.33
**P uptake effiency (mg P uptake mg^{-1} applied P)**						
P_{low}	0.44±0.06	0.01±0.00	0.12±0.07	0.34±0.06	0.01±0.00	0.06±0.04
P_{med}	0.14±0.02	0.02±0.01	0.17±0.00	0.18±0.01	0.15±0.01	0.12±0.02
P_{high}	0.09±0.01	0.06±0.01	0.08±0.00	0.10±0.00	0.09±0.02	0.12±0.01
**P use efficiency (g tuber DM mg^{-1} applied P)**						
P_{low}	0.23±0.06	-	0.15±0.05	0.16±0.01	-	-
P_{med}	0.11±0.02	0.03±0.02	0.13±0.00	0.11±0.01	0.10±0.01	0.05±0.02
P_{high}	0.07±0.00	0.04±0.00	0.07±0.00	0.07±0.00	0.08±0.01	0.03±0.01
**Residual soil P (mg g^{-1})**						
P_{low}	0.04±0.00	0.04±0.00	0.04±0.00	0.03±0.00	0.04±0.00	0.03±0.00
P_{med}	0.07±0.00	0.07±0.00	0.07±0.00	0.07±0.00	0.07±0.01	0.07±0.00
P_{high}	0.58±0.03	0.58±0.01	0.58±0.03	0.56±0.02	0.56±0.01	0.50±0.02

Phosphate concentrations is expressed as mg of the mineral (P) per unit of DM, dry matter.

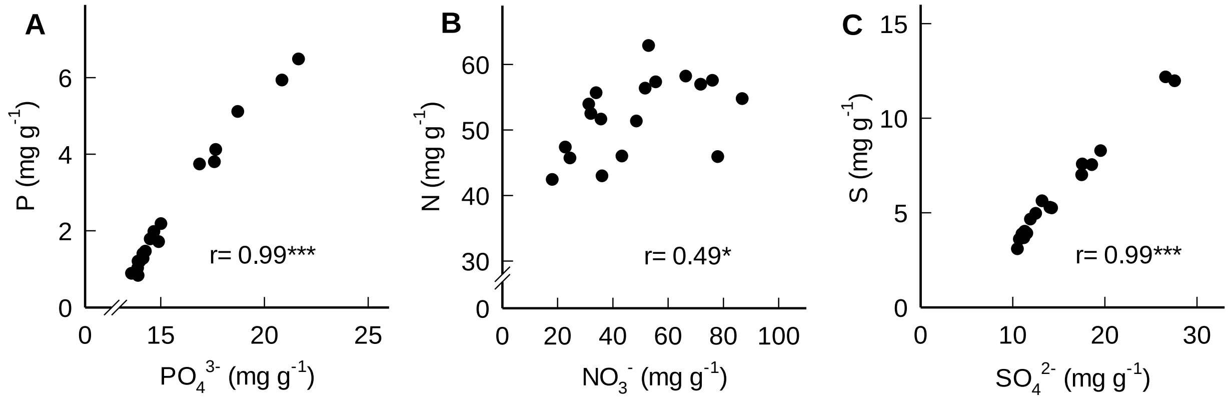

Supplementary Figure 2. Correlations between minerals and ions in young leaves at 53 DAE

Supplementary Table 5. Leaf mineral concentration (mg g^{-1} DM) of potato cultivars at 53 DAE under P_{low}, P_{med}, and P_{high} applications

	Cultivars					
	**Agria**	**Lady Claire**	**Lilly**	**Milva**	**Sieglinde**	**Verdi**
**C**						
P_{low}	384.04±3.61 b	355.05±5.79 a	342.96±9.38 b	381.19±0.87 a	354.00±3.48 b	368.03±9.12 a
P_{med}	417.17±2.77 a	388.57±9.45 a	404.71±9.09 a	403.27±9.10 a	427.10±9.40 a	391.91±9.05 b
P_{high}	430.07±1.70 a	381.38±5.19 a	405.25±4.12 a	410.19±9.91 a	426.46±3.66 a	405.14±6.90 b
**K**						
P_{low}	46.73±8.79 a	51.12±2.52 a	50.76±7.27 a	37.53±9.35 a	54.93±3.62 a	46.13±7.88 a
P_{med}	30.64±7.59 b	48.61±7.99 a	44.84±4.05 a	42.68±6.42 a	37.01±8.12 b	34.12±7.22 a
P_{high}	35.64±3.23 b	31.18±4.16 b	50.28±7.21 a	30.90±6.22 a	37.49±7.64 b	39.82±9.61 a
**Ca**						
P_{low}	23.71±2.24 a	42.31±2.82 a	34.15±6.14 a	21.30±0.51 a	28.68±3.33 a	36.45±3.09 a
P_{med}	17.31±2.52 a	43.99±3.39 a	25.65±2.06 a	20.08±0.89 a	22.38±1.27 a	22.39±3.20 b
P_{high}	20.90±2.74 a	26.13±2.46 b	28.72±3.70 a	20.45±1.40 a	20.82±1.87 a	18.88±1.15 b
**Mg**						
P_{low}	5.75±0.34 b	5.48±0.27 a	3.71±0.12 a	6.19±0.33 a	5.57±0.21 b	5.58±0.30 b
P_{med}	6.04±0.39 a	7.01±0.67 a	4.64±0.62 a	6.50±1.15 a	5.51±0.68 b	6.06±0.74 ab
P_{high}	6.66±1.48 a	6.25±0.69 a	4.84±0.35 a	6.84±0.93 a	7.17±0.95 a	6.56±1.28 a
**Mn**						
P_{low}	0.26±0.02 a	0.17±0.01 b	0.52±0.05 a	0.38±0.01 a	0.37±0.04 a	0.23±0.06 a
P_{med}	0.21±0.01 a	0.24±0.03 ab	0.30±0.03 b	0.18±0.01 b	0.29±0.03 a	0.19±0.04 a
P_{high}	0.26±0.04 a	0.36±0.04 a	0.38±0.03 ab	0.27±0.02 b	0.31±0.04 a	0.21±0.02 a
**Fe**						
P_{low}	0.15±0.01 a	0.14±0.02 a	0.17±0.03 a	0.18±0.06 a	0.19±0.01 a	0.13±0.01 a
P_{med}	0.17±0.02 a	0.16±0.02 a	0.16±0.01 a	0.15±0.01 a	0.14±0.01 b	0.14±0.03 a
P_{high}	0.17±0.00 a	0.13±0.01 a	0.17±0.02 a	0.16±0.01 a	0.14±0.00 b	0.12±0.01 a
**Zn**						
P_{low}	0.20±0.02 a	0.32±0.07 a	0.30±0.03 a	0.26±0.01 a	0.37±0.09 a	0.30±0.03 a
P_{med}	0.14±0.02 b	0.26±0.06 b	0.14±0.02 b	0.15±0.01 b	0.10±0.01 b	0.18±0.02 ab
P_{high}	0.14±0.01 b	0.16±0.02 c	0.15±0.01 b	0.16±0.01 b	0.13±0.00 b	0.13±0.02 b

Mean values ± SE (n=4) with different lowercase letters indicate significant difference between P treatments of each cultivar by Tukey's HSD test at $p<0.05$. DM, dry matter.

Supplementary Table 6. Means ±SE of leaf photosynthesis and biochemical properties

	Cultivars					
	**Agria**	**Lady Claire**	**Lilly**	**Milva**	**Sieglinde**	**Verdi**
**CO_2 assimilation rate at 35 DAE (μmol m^{-2} s^{-1})**						
P_{low}	6.75±2.57	1.62±0.50	1.47±0.64	4.84±0.85	-	1.29±0.40
P_{med}	12.41±1.77	7.50±1.95	14.15±1.46	15.92±1.02	5.12±1.18	7.42±2.41
P_{high}	8.49±4.72	12.03±1.91	10.13±1.93	12.82±2.73	11.92±2.52	11.30±1.61
**CO_2 assimilation rate at 53 DAE (μmol m^{-2} s^{-1})**						
P_{low}	2.76±0.46	6.16±2.47	1.54±0.04	4.29±1.11	2.89±0.70	4.79±1.00
P_{med}	14.47±1.75	11.77±0.33	11.53±0.23	13.35±4.87	7.87±5.24	3.04±2.91
P_{high}	7.97±0.58	12.32±4.13	11.53±1.35	9.20±0.88	10.66±4.53	11.82±0.39
**CO_2 assimilation rate at 70 DAE (μmol m^{-2} s^{-1})**						
P_{low}	7.71±1.99	-	-	9.94±4.61	-	4.40±2.32
P_{med}	12.28±1.00	13.27±2.00	11.80±0.98	11.42±0.85	5.83±1.16	10.46±2.03
P_{high}	10.24±3.15	11.57±1.93	13.66±1.01	7.83±1.43	10.00±2.16	12.16±2.02
**Stomatal conductance at 35 DAE (mmol m^{-2} s^{-1})**						
P_{low}	0.77±0.32	0.33±0.10	0.57±0.08	0.65±0.22	0.00±0.00	0.31±0.15
P_{med}	1.51±0.34	1.13±0.23	1.81±0.29	2.18±0.13	0.63±0.12	0.84±0.29
P_{high}	1.17±0.73	1.36±0.27	1.08±0.22	1.51±0.40	1.42±0.37	1.14±0.24
**Stomatal conductance at 53 DAE (mmol m^{-2} s^{-1})**						
P_{low}	0.53±0.02	0.99±0.62	0.45±0.04	0.82±0.14	0.59±0.10	0.90±0.20
P_{med}	2.65±0.60	2.08±0.47	2.53±0.29	2.18±0.51	1.44±0.38	3.46±2.88
P_{high}	1.54±0.25	1.85±0.37	1.98±0.61	1.60±0.04	1.75±0.76	1.98±0.06
**Stomatal conductance at 53 DAE (mmol m^{-2} s^{-1})**						
P_{low}	1.00±0.19	0.00±0.00	0.00±0.00	1.30±0.72	0.00±0.00	0.53±0.22
P_{med}	1.61±0.20	2.24±0.30	1.76±0.27	1.33±0.23	0.75±0.10	1.18±0.24
P_{high}	1.29±0.39	1.69±0.37	1.92±0.32	0.86±0.15	1.37±0.41	1.32±0.25
**Intercelluar CO_2 at 35 DAE (ppm)**						
P_{low}	182.83±3.84	282.07±15.00	326.70±34.73	204.10±20.66	-	306.76±30.00
P_{med}	202.02±21.37	243.46±26.79	220.94±11.37	231.55±3.30	206.34±10.49	186.24±6.38
P_{high}	211.24±17.47	198.39±6.14	175.93±6.72	191.71±20.63	194.24±15.86	161.98±14.86
**Intercelluar CO_2 at 53 DAE (ppm)**						
P_{low}	219.69±23.07	178.03±3.71	284.75±23.66	227.80±69.18	284.33±21.00	172.23±13.00
P_{med}	231.11±9.93	230.19±13.90	208.05±29.12	216.28±37.90	257.55±20.28	100.84±62.10
P_{high}	174.77±20.69	190.22±31.21	185.79±3.37	169.62±5.66	176.18±51.06	177.03±18.90
**Intercelluar CO_2 at 70 DAE (ppm)**						
P_{low}	220.38±7.90	-	-	207.46±24.55	-	257.77±57.03
P_{med}	215.05±11.00	263.23±12.00	241.96±10.05	190.91±20.31	217.06±23.82	189.83±5.13
P_{high}	212.76±19.12	230.02±15.75	226.86±16.94	182.70±2.86	211.89±22.56	178.63±8.75
**ATP (nmol g^{-1} FW)**						
P_{low}	10.56±3.74	5.52±1.23	3.68±0.58	2.25±0.80	18.67±4.28	7.45±4.12
P_{med}	22.11±4.34	13.41±5.85	13.85±2.40	24.50±7.32	16.40±3.02	13.58±4.98
P_{high}	45.93±8.26	25.43±6.48	35.25±9.29	31.27±4.90	44.58±6.53	25.38±4.34
**Protein (mg g^{-1} FW)**						
P_{low}	0.92±0.13	2.66±1.65	0.67±0.09	0.85±0.17	1.06±0.19	1.16±0.15
P_{med}	1.16±0.12	1.08±0.11	0.92±0.06	0.99±0.13	0.77±0.15	1.24±0.15
P_{high}	0.73±0.07	1.42±0.11	0.89±0.11	0.96±0.15	0.87±0.14	1.22±0.18
**Chlorophyll (mg g^{-1} FW)**						
P_{low}	1.25±0.07	0.97±0.04	0.93±0.05	0.87±0.29	0.90±0.06	1.11±0.11
P_{med}	1.30±0.03	0.95±0.05	1.03±0.06	1.28±0.02	1.20±0.02	1.50±0.09
P_{high}	1.26±0.06	1.11±0.03	1.05±0.09	1.18±0.05	1.26±0.12	1.41±0.10
**Proline (μmol g^{-1} FW)**						
P_{low}	5.18±0.77	6.88±0.97	8.35±0.49	4.95±0.38	9.80±1.85	7.74±1.12
P_{med}	2.20±0.36	8.19±0.54	5.22±0.74	4.07±0.62	6.95±0.48	6.64±1.20
P_{high}	2.15±0.45	4.46±0.72	3.86±0.97	2.64±0.72	3.62±0.29	4.18±0.93
**Sucrose (mg g^{-1} DM)**						
P_{low}	7.64±1.29	6.65±0.76	10.57±0.80	6.88±0.29	19.71±3.49	9.09±2.09
P_{med}	7.53±1.95	12.51±2.72	14.42±0.89	12.80±0.70	13.07±1.30	10.77±1.45
P_{high}	7.23±1.67	10.84±1.97	15.54±2.49	14.88±1.90	14.94±1.37	10.34±0.34
**Reducing sugar (mg g^{-1} DM)**						
P_{low}	5.16±0.38	2.77±0.72	2.55±0.71	3.05±0.45	25.50±9.39	3.92±0.63
P_{med}	21.37±4.56	5.39±0.81	29.93±6.16	29.80±5.86	20.95±5.49	10.73±2.85
P_{high}	38.41±7.20	11.65±1.81	44.89±6.88	36.48±1.60	47.41±3.86	23.93±5.80

Supplementary Table 7. Analysis of variance (ANOVA) of tuber quality of three potato cultivars under P_{low}, P_{med}, and P_{high}. FW, fresh weight; DM, dry matter.

	**ANOVA**		
	**Cultivar (C)**	**P levels (P)**	**C x P**
Dry matter	***	***	*
Starch	*	***	ns
Protein	***	***	ns
Soluble sugars	***	ns	ns
Ascorbic acid	ns	**	ns
TPC	*	***	**
TFC	ns	***	ns
DPPH	ns	***	ns
TEAC	*	***	*
Tuber minerals			
C	ns	***	ns
K	ns	***	ns
S	*	***	ns
Ca	**	***	**
Mg	*	***	**
Mn	**	***	ns
Fe	**	ns	ns
Zn	**	***	**
Tuber ions			
PO_4^{3-}	***	**	***
NO_3^-	***	**	*
SO_4^{2-}	***	ns	**

ns, *, **, and *** indicate non-significance and significance at p<0.05, p<0.01, and p<0.001, respectively.

Supplementary Table 8. Means±SE of nutritional quality parameters of tubers

	Cultivars		
	**Agria**	**Lilly**	**Miva**
**Dry matter (%)**			
P_{low}	15.30±0.20	14.43±0.67	14.59±0.63
P_{med}	19.33±0.26	18.62±0.43	21.39±0.44
P_{high}	21.43±0.22	19.58±0.48	21.55±0.44
**Starch (% DM)**			
P_{low}	53.41±0.83	51.26±1.71	53.77±2.10
P_{med}	62.16±1.07	62.22±1.08	62.75±1.05
P_{high}	69.70±0.92	63.56±1.21	65.91±1.05
**Protein (% DM)**			
P_{low}	16.11±0.77	20.39±1.13	15.01±0.54
P_{med}	12.72±0.99	13.10±0.71	9.10±0.38
P_{high}	9.41±0.86	11.03±0.80	8.44±0.38
**Soluble sugars (mg g^{-1} DM)**			
P_{low}	73.69±3.08	60.81±19.08	103.28±12.32
P_{med}	67.50±4.84	73.58±1.49	89.56±6.17
P_{high}	62.15±4.00	77.50±4.09	85.46±3.87
**Ascorbic acid (mg g^{-1} FW)**			
P_{low}	0.17±0.01	0.18±0.01	0.19±0.01
P_{med}	0.23±0.02	0.23±0.01	0.21±0.00
P_{high}	0.21±0.02	0.24±0.01	0.19±0.00
**TPC (mg g^{-1} DM)**			
P_{low}	0.96±0.09	1.34±0.17	1.46±0.03
P_{med}	1.06±0.05	0.82±0.03	1.05±0.11
P_{high}	0.85±0.05	0.78±0.05	0.92±0.03
**TFC (mg g^{-1} DM)**			
P_{low}	0.53±0.05	0.61±0.01	0.50±0.04
P_{med}	0.48±0.08	0.50±0.02	0.50±0.03
P_{high}	0.39±0.05	0.27±0.02	0.24±0.01
**DPPH (µmol g^{-1} DM)**			
P_{low}	6.43±1.19	6.67±0.30	8.82±1.07
P_{med}	4.51±0.42	3.88±0.18	4.28±0.29
P_{high}	4.31±0.81	3.86±0.47	3.77±0.16
**TEAC (µmol g^{-1} DM)**			
P_{low}	6.03±0.75	6.61±0.12	9.38±0.90
P_{med}	5.32±0.28	4.73±0.58	4.99±0.34
P_{high}	4.71±0.39	4.87±0.46	4.93±0.13

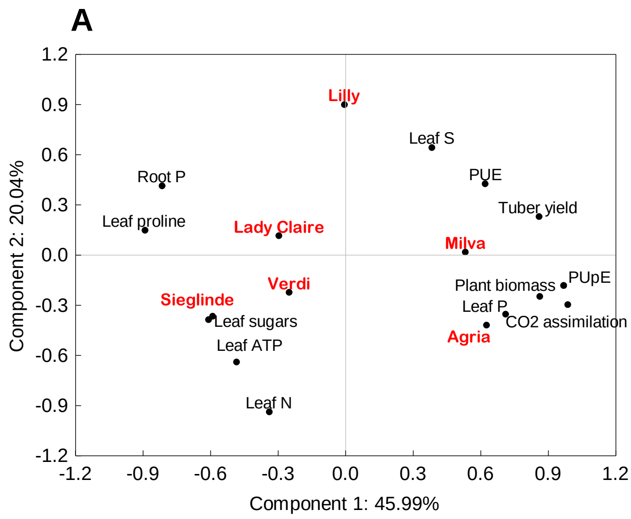

Supplementary Figure 3. Principal component analysis of traits associated with P efficiency of cultivars under (A) P_{low} and (B) P_{high} conditions. The leaf parameters such as P, S, N, proline, sugars, ATP, and CO2 assimilation rate were taken at 53 DAE. Root P, plant biomass, tuber yield, PUpE, and PUE were measured on plants at 87 DAE. PUpE= P uptake efficiency, PUE= P use efficiency.
